# Spontaneous instrumental avoidance learning in social contexts

**DOI:** 10.1038/s41598-022-22334-6

**Published:** 2022-10-20

**Authors:** Rocco Mennella, Sophie Bavard, Inès Mentec, Julie Grèzes

**Affiliations:** 1 Laboratoire des Interactions Cognition, Action, Émotion (LICAÉ), Université Paris Nanterre, 200 Avenue de La République, 92001 Nanterre Cedex, France; 2 Cognitive and Computational Neuroscience Laboratory (LNC2), Inserm U960, Department of Cognitive Studies, École Normale Supérieure, PSL University, 29 Rue d’Ulm, 75005 Paris, France; 3 Department of Psychology, University of Hamburg, Von-Melle-Park 11, 20146 Hamburg, Germany

**Keywords:** Human behaviour, Decision, Motivation, Reward, Emotion

## Abstract

Adaptation to our social environment requires learning how to avoid potentially harmful situations, such as encounters with aggressive individuals. Threatening facial expressions can evoke automatic stimulus-driven reactions, but whether their aversive motivational value suffices to drive instrumental active avoidance remains unclear. When asked to freely choose between different action alternatives, participants spontaneously—without instruction or monetary reward—developed a preference for choices that maximized the probability of avoiding angry individuals (sitting away from them in a waiting room). Most participants showed clear behavioral signs of instrumental learning, even in the absence of an explicit avoidance strategy. Inter-individual variability in learning depended on participants’ subjective evaluations and sensitivity to threat approach feedback. Counterfactual learning best accounted for avoidance behaviors, especially in participants who developed an explicit avoidance strategy. Our results demonstrate that implicit defensive behaviors in social contexts are likely the product of several learning processes, including instrumental learning.

## Introduction

Imagine that you have started a new job and you have noticed that a new colleague is often angry. To stay out of trouble, you will rapidly learn what you must do to avoid meeting him at the coffee machine or, at least, to sit as far as possible from him in the cafeteria. Adaptation to our complex social environment requires the ability to flexibly learn how to avoid or escape potentially harmful situations, such as encountering aggressive individuals. Active avoidance is one peculiar kind of defensive behavior, “in which subjects learn to minimize or prevent contact with aversive events […] by taking action”^1^. Active avoidance has classically been studied through conditioning paradigms built onto two learning phases, a Pavlovian and an instrumental phase e.g.,^1–4^. First, via Pavlovian conditioning, a neutral stimulus (CS +) is associated with an aversive outcome (US, often an electrical shock). Second, because the CS + allows individuals to predict the occurrence of an aversive event, they learn to perform specific actions to avoid the US (instrumental conditioning). Depending on the paradigm, such instrumental (goal-directed) actions can either prevent the occurrence of the US after CS + presentation (primary avoidance or US-avoidance) or directly prevent the CS + (secondary avoidance or CS-avoidance). In variations of active avoidance paradigms, subjects learn not to prevent, but to terminate—or increase the distance in space/time with—either the CS + (threat escape or CS-escape) or the ongoing US (harm-escape or US-escape)^5,6^. Despite some interesting psychophysiological differences^7,8^, both active avoidance and escape are acquired through the same biphasic learning procedure and share neural substrates^9^. They are therefore commonly considered variations of instrumental active avoidance responses^10^.

Human experiments have mainly focused on US- avoidance e.g.,^11–14^, while less is known on CS-avoidance but see^6–8,15,16^. This is surprising, given that, in social settings, we often begin avoiding the new aggressive colleague before experiencing any concrete negative consequences. Individuals learn throughout life that angry facial displays, which communicate the intent to inflict verbal and/or physical harm, are a danger-predictive cue (a CS +), either by Pavlovian conditioning^17–19^ or by experience and observation e.g.,^20,21^. Therefore, previous experience informs us that angry expressions might function as ready-to-go signals that help us predict and avoid possible future negative consequences. The aversive motivational value of angry expressions is evidenced through their associated perceptual and attentional advantages e.g.,^22–26^, induced physiological changes^27,28^ as well as their influence on the anticipatory processing of action intentions^29^ and action selection^30–32^, even in the absence of awareness^33^. To our knowledge, while the influence of social threat displays on specie-specific defensive behaviors, such as freezing^34^, or on stimulus-driven avoidance tendencies^35–37^ has been established, it remains unknown as to whether they can drive instrumental CS-avoidance learning.

Here, we first tested the possibility that the aversive motivational value of angry facial displays can mediate instrumental CS-avoidance learning. We hypothesized that participants would learn to select, from among two options, the action that maximizes the probability of increasing the spatial distance from a threatening individual (avoidance) in a subsequent feedback phase. That is, consistently with previous results on humans showing that successful avoidance is intrinsically rewarding^38^, we propose that the outcome “avoiding anger” is preferred to “approaching anger”, and, as such, will act as a reinforcer to promote instrumental CS-avoidance learning. To test our hypothesis, we converted our original free choice paradigm^30–32^ into a reinforcement-learning (RL) task. Standard RL tasks require subjects to explicitly maximize their reward, often in terms of maximising points/money gains or minimising points/money losses. To do so, subjects need to learn, in a preliminary Pavlovian phase, which option was associated with the best outcome. However, our tasks did not use monetary or point incentives but relied instead entirely on the intrinsic motivational value of the socio-emotional outcome of an action and, consequently, did not include a preliminary Pavlovian phase.

**Figure 1:**
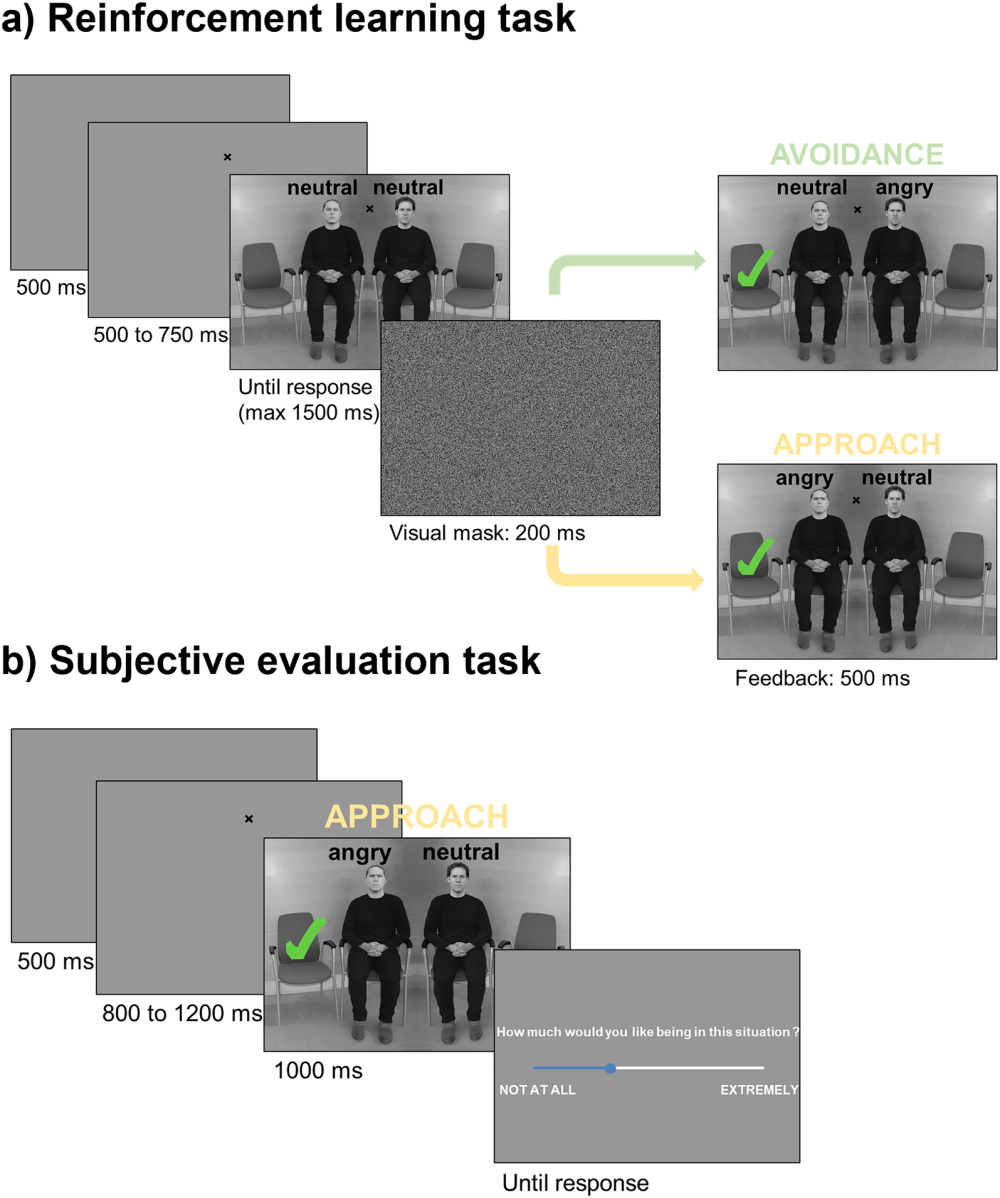
Behavioral tasks. (**a**) In the reinforcement learning task, participants indicated on which free chair they would like to sit, via a left/right button press. The figure shows an example trial, in which the participant pressed the left button. In the feedback phase he could find himself far from (avoidance, in green) or close to (approach, in yellow) the individual displaying anger, depending on a hidden probability associated with each chair. (**b**) In the subjective evaluation task that followed the reinforcement learning task, participants had to subjectively evaluate each possible scenario seen in the feedback phase of the previous task (the example is of an approach scenario). Please note that the identities displayed were taken from the Radboud Faces Database and were selected for illustration purposes only (they were not used for the real experiment), following the guidelines of the database.

Two samples of subjects (pilot n = 62, main experiment n = 278) were presented with a waiting room with four chairs, where the two middle chairs were occupied by two individuals and the two outer chairs were empty (See Fig. 1a). Subjects were instructed to indicate via button press on which chair in the scene they would prefer to sit, knowing that no wrong/right responses existed. At choice time, both individuals had a neutral expression but, immediately following the subject’s response, one of the individuals changed expression from neutral to anger, for 500 ms. Accordingly, in this short feedback phase following choice, subjects would find themselves either far from (avoidance scenario) or close to (approach scenario) the individual expressing anger. At each trial, one response (e.g., sit on the right chair) was associated with an 80% probability of avoidance in the feedback phase and to a 20% probability of approach. The other response (e.g., sitting on the left chair) was associated with the complementary probabilities (20% avoidance, 80% approach). Therefore, in our task, one angry individual was always present in the feedback phase, while the subject's physical distance from the angry individual changed depending on response choice, similarly to threat escape paradigms. Furthermore, presenting the full scene in the feedback phase allowed for counterfactual learning, which refers to the ability to learn from forgone outcomes, i.e., the unchosen option^39^, which has proven to be of importance in avoidance learning e.g.,^40^. To discourage habit formation or simple response perseverance, which play important roles in the instantiation of non-goal-directed avoidance responses^1^, and to ensure continuous instrumental learning, we introduced volatility in our environment, by setting action-outcome probability reversals every 25 trials on average.

Instrumental learning in humans and other animals relies on both implicit and explicit predictions of action outcomes^10,41,42^. Most experiments have investigated explicit active avoidance, where individuals were told that performing a certain action would allow them to avoid the US or CS e.g.,^11,43,44^, including studies using emotional faces^45^. In contrast, we never mentioned the presence of threatening facial displays to the participants, nor that one choice would be associated with a higher probability of avoiding the threatening individual. We expected that, thanks to the experience accumulated across trials, the available action options would acquire positive or negative experiential values from past outcomes, i.e., virtually sitting away from or next to an angry individual, respectively. Therefore, we predicted that participants would develop a preference for choosing the action with the highest subjective value, i.e., sitting on the chair that allowed them to avoid the angry individual, similarly to what might happen in real-life situations. Furthermore, as both human and non-human animal studies have observed large individual differences in US-avoidance learning and behavior e.g.,^12,46,47^, we investigated, on the basis of our pilot results and via an extensive debriefing at the end of the experiment, whether such individual differences in instrumental CS-avoidance learning could be sourced to the participant’s ability to explicitly report having developed an avoidance strategy. Finally, to further understand whether a spontaneous emergence of avoidance was linked to the subjective value that each subject attributed to approach/avoidances scenarios, we ran a short subjective evaluation task after the reinforcement learning task, asking for an explicit subjective evaluation of each possible scenario encountered in the main task (See Fig. 1b). We predicted that subjects would subjectively prefer avoidance vs. approach scenarios, and that this preference would account for the spontaneous emergence of avoidance behavior in the main task.

## Results

### Social threat avoidance emerges spontaneously via instrumental learning

To test whether subjects spontaneously learned to avoid the angry individual, we first ran mixed logistic models on the probability of “hits”, namely the proportion of trials in which participants chose to sit on the chair that maximized the probability of avoidance in the feedback phase.

**Figure 2:**
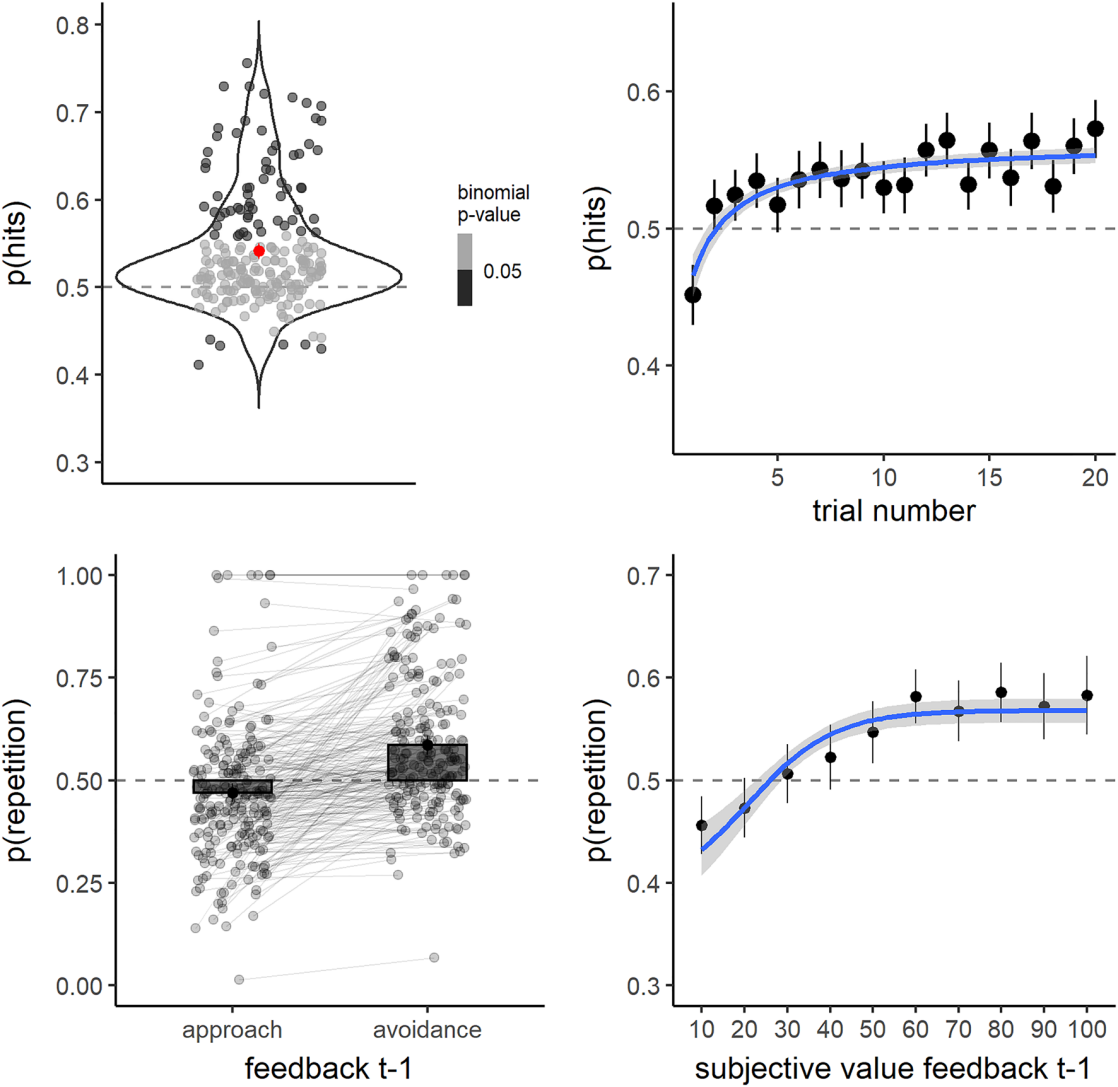
Summary of behavioral results. Left top: proportion of hits throughout the task. The red point represents the mean while the error-bar represents the confidence interval at 95% for the normal distribution. Shaded points represent single subject means while grey tones reflects whether, within each subject, the binomial test against chance (0.5) is significant (dark grey) or not (light grey). Right top: mean proportion of hits across the first 20 trials over blocks of stable action-outcome contingency (trial 1 = reversal trial). Points represent means within trial and error-bars represent confidence intervals at 95% for the normal distribution. The fitted curve represents the best fit (and 95% confidence interval) for the same hyperbolic function used in the mixed linear models (see “Methods”). Left bottom: mean proportion of action repetition following either approach or avoidance feedback. Black points represent means while error-bars represent confidence intervals 95% for the normal distribution. Shaded points represent single subject means. Right bottom: mean proportion of action repetition as a function of the subjective value attributed to the feedback obtained at t − 1. Black points represent means while error-bars represent confidence intervals 95% for the normal distribution. The fitted curve represents the best fit (and 95% confidence interval) for a logistic function.

The first model included the proportion of hits as a dependent variable and the intercept as a predictor, measuring difference from chance^48^ (see “Statistical analyses” for full details on the fixed and random effects’ structure). Average hit rate was higher than chance level 0.5, as indicated by the significant fixed effect of the intercept (Odd Ratio (OR) = 1.18, OR_IC95%_ = 1.14 − 1.23, *p* < 0.001; see Table 1, model 1; Fig. 2 left top), signaling significant instrumental learning at the group level. Results from our second model, which included the effect of trial number within blocks of stable action-outcome contingency, showed that, on average, on the first trial after reversal hit rate fell significantly below chance (Intercept: OR = 0.83, OR_IC95%_ = 0.77 − 0.90, *p* < 0.001; see Table 1, model 2; Fig. 2 right top) to then increase hyperbolically above chance across the first twenty trials within a block of stable action-outcome contingencies (OR = 1.51, OR_IC95%_ = 1.35 − 1.70, *p* < 0.001; see Table 1, model 2; Fig. 2 right top). These results indicated that, across trials, subjects learned the association between each possible action and the respective outcome and updated this association after each reversal.

**Table 1:** Mixed logistic models.

	Model 1: hits			Model 2: hits			Model 3:repetition			Model 4:repetition		
Predictors (across models)	Odds ratios	CI	*p*	Odds ratios	CI	*p*	Odds ratios	CI	*p*	Odds ratios	CI	*p*
Intercept	1.18	1.14–1.23	** < 0.001**	0.83	0.77–0.90	** < 0.001**	0.93	0.82–1.07	0.304	0.85	0.75–0.96	**0.012**
Hyp(trial)				1.51	1.35–1.70	** < 0.001**						
Feedback t − 1 (avoidance)							1.73	1.56–1.93	** < 0.001**			
Subjective value feedback t − 1										2.50	2.09–2.98	** < 0.001**
**Random effects**												
σ^2^	3.29			3.29			3.29			3.29		
τ_00_	0.06_subject_			0.08_subject_			0.95_subject_			0.78_subject_		
τ_11_				0.35_subject.hyp(trial)_			0.55_subject.feedback t-1 (avoidance)_			1.33_subject.subjective value feedback t −1_		
ρ_01_				− 0.98_subject_			− 0.29_subject_			− 0.21_subject_		
ICC	0.02			0.02			0.24			0.23		
N	214_subject_			214_subject_			214_subject_			214_subject_		
Observations	62,815			50,240			62,093			62,093		
Marginal R^2^/Conditional R^2^	0.000/0.017			0.002/0.022			0.017/0.250			0.017/0.242		

Random effects: σ^2^ = Residual variance, τ_00_ = random intercept variance, τ_11_ = random slope variance, ρ_01_ = random intercept/slope correlation, ICC = Intraclass Correlation Coefficient.

Significant values are in bold.

To further characterize learning, we were interested in knowing whether subjects learned preferentially from positive/safety outcomes (avoidance) or negative/threat outcomes (approach). To this end we fitted a model predicting the probability of repeating at trial t the same action produced at trial t − 1, as a function of the obtained feedback (avoidance or approach). Interestingly, while the probability to repeat did not differ significantly from chance following approach feedback (Intercept: OR = 0.93, OR_IC95%_ = 0.82 − 1.07, *p* = 0.304; see Table 1, model 3; Fig. 2 left bottom), it increased significantly after avoidance feedback (OR = 1.73, OR_IC95%_ = 1.56 − 1.93, *p* < 0.001; see Table 1, model 3; Fig. 2 left bottom), suggesting that subjects learn preferentially from positive, as opposed to negative, outcomes.

Nonetheless, each subject might assign a very different value to each scenario, rendering it difficult to unambiguously attribute a positive value to avoidance feedback and a negative value to approach feedback. To check whether subjective values differed for approach vs. avoidance scenarios, we ran a mixed linear model on subject responses on the subjective evaluation task. Subjects evaluated how much they would have liked to find themselves in the proposed scenario, on a visual analog scale ranging from ‘NOT AT ALL’ to ‘EXTREMELY’, and responses were converted to a 0–100 scale. Results indicated that approach scenarios were rated on average at 25.34 (IC_95%_ = 23.24 − 27.44), while subjective value increased significantly by 37.05 (IC_95%_ = 33.97 − 40.13, *p* < 0.001) for avoidance as compared to approach scenarios (see Supplementary Material, Table S1, model 1; Fig. S1, left). Therefore, on average, subjects preferred to find themselves in an avoidance, as compared to an approach scenario. On this basis, we ran a mixed logistic model on the probability of response repetition, this time including the subjective value attributed in the subjective evaluation task to each feedback obtained at trial − 1 in the reinforcement learning task. Consistently with the previous model, higher subjective values attributed to the feedback at trial − 1 predicted an increase in the probability of repetition at trial t (OR = 2.50, OR_IC95%_ = 2.09 − 2.98, *p* < 0.001; see Table 1, model 4; Fig. 2 right bottom), an effect especially driven by an increase in the probability of repetition for positively-valued feedback (Fig. 2 right bottom).

### Controlling for the development of an explicit avoidance strategy

Figure 2 highlights substantial variability in learning, with some subjects clearly above chance level and others not very much so. Before running the present task, we ran an online pilot study on 62 subjects (6 excluded, see Supplementary Methods), where we began investigating potential sources of variability in a post-task debriefing, by asking participants whether they thought any feature of the faces influenced their choice (“Did you notice anything about the faces of the individuals in the scenes? Did this influence your choice? If so, how?”). We realized that approximately one third of subjects not only saw the emotion on the face in the feedback phase but reported strategically trying to avoid it (even if not all of them understood that there was a specific contingency between their response and the obtained outcome) (N_non-explicit_ = 35, N_explicit_ = 21). Roughly the same proportion of subjects, with an explicit versus non-explicit avoidance strategy, were found in the main experiment (N_non-explicit_ = 154, N_explicit_ = 60), confirming that only a minority were able to develop such an explicit strategy. Examples of subjects’ reports were: “If they looked angry after I sat next to them, I would sit on the opposite side the next time” or “If the expression was angry I selected the other option in the next round” (i.e., explicit avoidance strategy) and “I don’t think it consciously influenced my choice because there was so little time to think, only to click”, “yes but I cannot explain why” or simply “no” (i.e., non-explicit strategy) (see Supplementary Material, Table S2 for full subjects' report).

**Figure 3:**
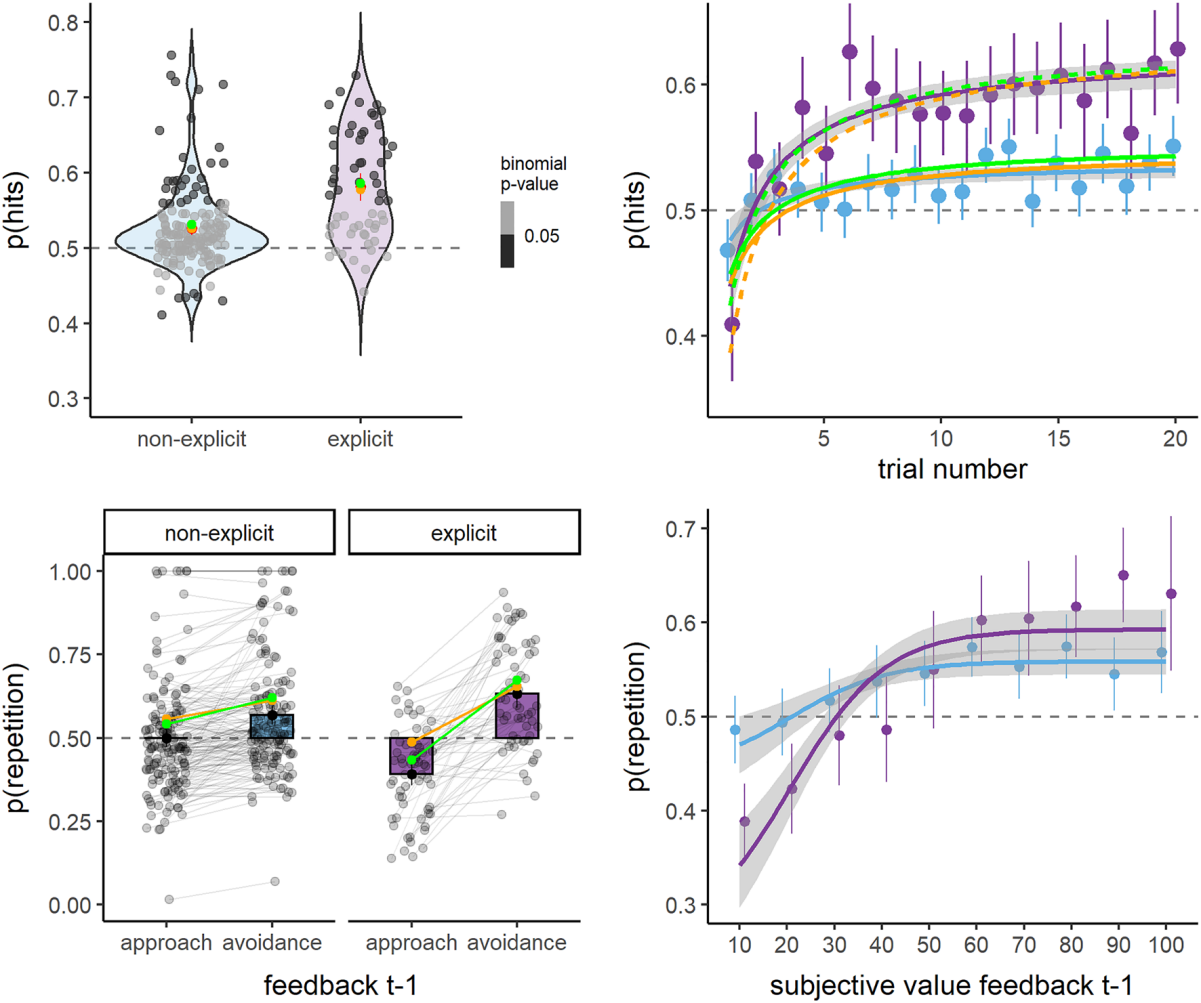
Summary of behavioral results as a function of the presence/absence of an explicit avoidance strategy. All information provided in the legend for Fig. 2 applies to Fig. 3. The figure highlights the differences between the group without an explicit avoidance strategy (light blue) and the group with an explicit strategy (violet). Prediction from the simple and the counterfactual reinforcement learning models (see next paragraph and “Methods”) are in orange and green, respectively (solid lines for the group without an explicit strategy and dotted lines for the one with explicit strategy). Orange and green points represent means of simulations, and the fitted curve in the right top graph represents the best fit for the same hyperbolic function used in the mixed linear models, fitted on simulated data.

Therefore, we re-ran all previous models, including the main effect of strategy (0 = non-explicit, 1 = explicit) as a between-subject variable, as well as the interaction of strategy with the other variables (Table 2; Fig. 3). Results showed that the development of an explicit strategy was associated with better avoidance learning, as indicated by higher hit proportion (Strategy: OR = 1.25, OR_IC95%_ = 1.16–1.35, *p* =  < 0.001; see Table 2, model 1; Fig. 3 left top) and a steeper hyperbolic increase of hits across trials within blocks (hyp(trial)*strategy: OR = 1.74, OR_IC95%_ = 1.36–2.22, *p* < 0.001; see Table 2, model 2; Fig. 3 right top). Most interestingly, instrumental learning for the non-explicit strategy group remained significant, as indicated by the model 1 intercept (OR = 1.11, OR_IC95%_ = 1.07–1.16, *p* < 0.001; see Table 2, model 1; Fig. 3 left top) and by the model 2 true main effect of hyperbolic increase of hits across trials within blocks (hyp(trial): OR = 1.30, OR_IC95%_ = 1.14–1.47, *p* < 0.001; see Table 2, model 2; Fig. 3 right top). Furthermore, model 3 indicated that, while individuals without an explicit strategy did not differ significantly from chance in the probability of repeating the previous response following approach feedback (Intercept: OR = 1.09, OR_IC95%_ = 0.94–1.27, p = 0.258), they did repeat more often the previous response after avoidance feedback (feedback t-1: OR = 1.40, OR_IC95%_ = 1.25–1.57, *p* < 0.001; see Table 2, model 3; Fig. 2 left bottom). Individuals with an explicit strategy learned both from approach and avoidance feedback (feedback t − 1 * strategy: OR = 2.14, OR_IC95%_ = 1.73–2.65, *p* < 0.001; see Table 2, model 3; Fig. 2 left bottom). Model 4 confirmed that subjective value associated with the received feedback was also a significant predictor of the subsequent response in the group without an explicit strategy (subjective value feedback t − 1: OR = 1.79, OR_IC95%_ = 1.48–2.16, *p* < 0.001), and that the interaction with the factor strategy was significant as in model 3 (subjective value feedback t − 1 * strategy: OR = 3.10, OR_IC95%_ = 2.20–4.38, *p* < 0.001; see Table 2, model 4; Fig. 2 right bottom). At the subjective level, subjects with an explicit strategy valued as less positive the proposed scenarios overall (strategy: Estimate = − 7.45, IC_95%_ = − 12.02 to − 2.88, *p* < 0.001). Despite the fact that this effect appeared to be mostly driven by a difference in the approach condition (see Supplementary Material, Fig. S1), the interaction term did not reach significance (*p* = 0.146).

**Table 2:** Mixed logistic models.

	Model 1: hits			Model 2: hits			Model 3:repetition			Model 4:repetition		
Predictors (across models)	Odds Ratios	CI	*p*	Odds Ratios	CI	p	Odds Ratios	CI	*p*	Odds Ratios	CI	*p*
Intercept	1.11	1.07–1.16	**< 0.001**	0.88	0.81–0.97	**0.008**	1.09	0.94–1.27	0.258	1.01	0.87–1.16	0.922
Strategy (explicit)	1.25	1.16–1.35	** < 0.001**	0.80	0.67–0.95	**0.010**	0.57	0.43–0.76	** < 0.001**	0.57	0.43–0.74	** < 0.001**
Hyp(trial)				1.30	1.14–1.47	** < 0.001**						
Hyp(trial) * strategy (explicit)				1.74	1.36–2.22	** < 0.001**						
Feedback t − 1 (avoidance)							1.40	1.25–1.57	** < 0.001**			
Feedback t − 1 (avoidance) * strategy (explicit)							2.14	1.73–2.65	** < 0.001**			
subjective value feedback t − 1										1.79	1.48–2.16	** < 0.001**
subjective value feedback t − 1 * strategy (explicit)										3.10	2.20–4.38	** < 0.001**
**Random effects**												
σ^2^	3.29			3.29			3.29			3.29		
τ_00_	0.05_subject_			0.07_subject_			0.88_subject_			0.74_subject_		
τ_11_				0.29_subject.hyperbole(trial_num)_			0.43_subject.rew_prec_factavoidance_			1.01_subject.subjValue_prec_		
ρ_01_				− 0.98_subject_			− 0.19_subject_			− 0.11_subject_		
ICC	0.01			0.02			0.23			0.22		
N	214_subject_			214_subject_			214_subject_			214_subject_		
Observations	62,815			50,240			62,093			62,093		
Marginal R^2^/Conditional R^2^	0.003/0.017			0.006/0.022			0.024/0.249			0.022/0.241		

Controlling for the effect of having an explicit strategy.

Random effects: σ^2^ = Residual variance, τ_00_ = random intercept variance, τ_11_ = random slope variance, ρ_01_ = random intercept/slope correlation, ICC = Intraclass Correlation Coefficient.

Significant values are in bold.

To conclude, results from mixed models indicated that: (1) on aggregate, subjects showed significant instrumental learning based on the approach/avoidance feedback provided; (2) avoidance scenarios were associated, on average, with a more positive subjective value than approach scenarios, which affected learning; (3) subjects who developed an explicit avoidance strategy during the task were better at learning how to avoid the threatening individual and valued the proposed scenarios as more negative, in general. Furthermore, they learned both from approach (negative) and avoidance (positive) outcomes, contrary to subjects without an explicit avoidance strategy who seemed to learn only from avoidance (positive) outcomes. Concerning the replicability of these behavioral results, the results from our pilot study align largely to those of the main task (see Supplementary Material, Fig. S2; Table S3; note that the pilot study did not include a subjective evaluation task).

### Avoidance learning is counterfactual

Previous research has shown learning advantages when one’s choices and their associated outcomes are considered in the context of alternative outcomes that might have been experienced from alternative but unchosen options^39^. In the present task, we reasoned that the subjects may or may not integrate, within their model of the environment, that the two possible action options had a complementary probability of leading to an approach or an avoidance outcome. To test this, we contrasted reinforcement learning (RL) models^49,50^ where only the value of the chosen option was updated after each choice (simple learning), to models where both the value of the chosen and the unchosen option were updated (counterfactual learning), with the unchosen option being updated based on the value of the complementary feedback to the feedback obtained (see “Methods”). Furthermore, we compared these models to a random model giving random choices (50% probability of choosing right or left), to check whether each model fit the data better than chance. Due to the peculiarity of this task, where the action choice which maximizes reward (i.e., threat avoidance) remained the same over the course of an entire block before probability reversal, both the simple and the counterfactual models were slightly complexified versions of the classic RL model, to account for the tendency to repeat actions previously performed^51^. Other than the learning rate (α) and the inverse temperature (β) parameters, they both integrated a learning parameter α_hab_ that captured that tendency of subjects to value an action on the basis on how many times it had been chosen in the past (see “Methods”). We acknowledge that in our task an actual habit would probably not have had the time to develop over the course of a block, therefore α_hab_ can be considered a simple measure of response perseveration, i.e., the tendency to repeat previously performed responses. The instrumental action value Q and the "perseverance" value H were weighted by a final parameter w, which indicated how much a subject was prone to respond based on the tendency to persevere (values tending to 1) or on the instrumental value of the action (values tending to 0; for more details see “Methods”).

**Figure 4:**
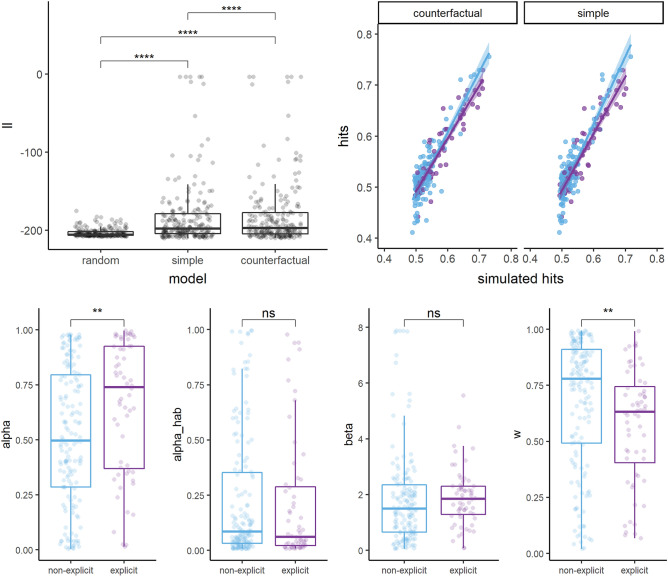
RL models results. Left top, log-likelihood comparison (Wilcoxon paired tests) between the random, simple, and counterfactual models. Right top, correlation between real and simulated mean hit proportions for the simple and the simulated models, as a function of the presence (violet) or the absence (light blue) of an explicit avoidance strategy. Lines represent the best linear fit (and 95% Confidence Interval). Bottom, comparison (Wilcoxon unpaired tests) between the groups with (violet) and without (light blue) an explicit avoidance strategy on subject best fit parameters for the winning counterfactual model. *=  *p* < .05, **= *p* < .01, ***= *p* < .001.

The Friedman test on RL model log-likelihood was statistically significant, χ^2^_(2)_ = 78.8, *p* < 0.001, indicating a difference in the ability of the models to fit the data (Fig. 4, left top). Wilcoxon paired tests, Bonferroni corrected, further indicated that the random model (median = − 206, min = − 208, max = − 175) had a lower likelihood compared to both the simple (Z = 2613, p_corr_ < 0.001, effect size = 0.67; median = − 198, min = -210, max = − 3.87) and the counterfactual models (Z = 2432, p_corr_ < 0.001, effect size = 0.68; median = − 197, min = − 210, max = − 3.88), which better fit the data. Importantly, the counterfactual model had a higher likelihood compared to the simple model (Z = 6638, p_corr_ < 0.001, effect size = 0.37). These results indicate that counterfactual models fitted the data significantly better than chance as well as better than the simple model (moderate effect size). Further investigation as to whether the counterfactual model better explained the data compared to the simple model within each group, demonstrates that this was the case for both the group without an explicit strategy (Z = 4270, p_corr_ = 0.007, effect size = 0.25, small) and with an explicit strategy (Z = 262, p_corr_ < 0.001, effect size = 0.62, large).

Both models reproduced the data very well, as indicated by the Spearman correlation between the simulated and the real hits; ρ = 0.81, *p* < 0.001 for the simple model and ρ = 0.82, *p* < 0.001 for the counterfactual model. For both models, the correlation was stronger for the group with an explicit strategy than for the one without (simple model: strategy ρ = 0.93, *p* < 0.001, no strategy ρ = 0.70, *p* < 0.001; counterfactual model: strategy ρ = 0.92, *p* < 0.001, no strategy ρ = 0.72, *p* < 0.001; Fig. 4, right top). Further visual appreciation of the models' ability to reproduce behavioral effects can be found in Fig. 3. Concerning the probability to repeat the previous action as a function of the obtained feedback (Fig. 3, left bottom), the counterfactual model (in green), contrary to the simple model (in orange), was able to reproduce the pattern for which the group without an explicit strategy learns primarily from avoidance (positive) feedback, and for which the group with an explicit strategy learns from both avoidance and approach feedback. Computationally, based on the structure of our models, the encoded value of each option is composed of a Q-value component (Q) and a perseverance (H) component. If the feedback of the chosen option is avoidance (i.e., positive feedback), then both components will be updated to overall favor the chosen option. However, if the feedback is approach (i.e., negative feedback), the Q-learning component will be decreased but the perseverance component will still be increased, which allows the model to account for the observed behavior. This insight is also consistent with the parameter values reported in Fig. 4 (bottom): for participants with an explicit strategy, the update of the Q-value is higher than for non-explicit participants because the learning rate is significantly higher. Hence, this could explain why the model is able to capture both (1) the tendency to learn more from one type of feedback than the other and (2) the difference in patterns between participants. The effect is even stronger for the counterfactual model because the Q-value updating following approach feedback goes even further in the opposite direction as to the habit update.

For the winning counterfactual model, we compared, using Wilcoxon tests, model parameters between the groups with and without an explicit avoidance strategy. Compared to the group without a strategy, the group with an explicit strategy had both a higher learning rate (median α_non-explicit_ = 0.50, median α_explicit_ = 0.74; Z = 3411, *p* = 0.003) and a stronger tendency to take into account the instrumental value of the action options, compared to their tendency to persevere (median w_non-explicit_ = 0.78, median w_explicit_ = 0.63; Z = 5917, *p* = 0.001). The two groups did not differ on the two remaining parameters (median α_hab_non-explicit_ = 0.084, median α_hab_explicit_ = 0.06; Z = 4995, *p* = 0.36; median β_non-explicit_ = 1.50, median β_explicit_ = 1.84; Z = 3928, *p* = 0.09).

### The more negative the value for threat approach, the better the instrumental avoidance learning

Table 3 presents the correlation between subject parameters for the winning counterfactual model and subjective measures, including both affective and personality questionnaires, and the mean subjective value attributed by each subject to approach and avoidance scenarios in the subjective evaluation task. Interestingly, the subjective value attributed by subjects to approach scenarios correlated with all parameters, except for β. In particular, the more negatively subjects valued approaching a threatening individual, the greater their learning rate α (ρ = − 0.2, p_unc_ = 0.004), as well as their propensity to use the instrumental value of the action to respond, as opposed to the simple tendency to persevere (w, ρ = 0.33, p_unc_ < 0.001). Consistently, subjects that tended to give a less negative evaluation of the approach scenario relied more on response perseverance (α_hab_, ρ = 0.16, p_unc_ < 0.022). At the same time, the value attributed to the avoidance (safe) scenario correlated with the β parameter, in the sense that the more positive the value the stronger the propensity to explore new action possibilities (ρ = − 0.18, p_unc_ = 0.009). Interestingly, within group correlations with α, w and β were stronger overall in the group without an explicit strategy as compared to the group with a strategy (see Supplementary Material, Tables S4 and S5). Concerning the questionnaires, across groups, neither anxiety nor autism traits appeared to be related to any of the learning parameters. A small negative correlation emerged between the Behavioral Inhibition System, and the w parameter (ρ = − 0.16, p_unc_ = 0.02), possibly indicating that the more the subjects were sensitive to negative and anxiogenic stimuli, the more they relied on the instrumental value of the response action, as opposed to the simple tendency to persevere. It must be noted that, when applying FDR correction for multiple comparisons, only the correlation between subjective value in the approach scenarios and w (p_corr_ < 0.001) and, marginally, α (p_corr_ = 0.08), remained significant.

**Table 3:** Spearman’s correlations between counterfactual model parameters and subjective measures.

	SVapp	SVav	STAIt	STAIs	BIS	BASd	BASr	BASf	BAPQa	BAPQpl	BAPQr
alpha	− 0.2**	0.02	0.05	0.08	0.06	− 0.01	− 0.01	0.02	− 0.09	− 0.11	− 0.04
alpha_hab_	0.16*	0.05	0	− 0.04	− 0.06	− 0.05	0.03	0.02	0.02	0.09	0.06
beta	0	− 0.18**	0.01	0.07	0.04	0.04	0	0.04	0.05	0.04	− 0.03
w	0.33***	− 0.02	− 0.11	− 0.04	− 0.16*	0.13	0.01	0.02	− 0.01	− 0.03	− 0.11

Correlations in bold survive FDR correction for multiple comparisons.SVapp = subjective value for approach scenarios, SVav = subjective value for avoidance scenarios, BIS = Behavioral Inhibition System, BASd = Behavioral Activation System, Drive subscale, BASr = Behavioral Activation System, Sensitivity to Reward subscale, BASd = Behavioral Activation System, Funseeking subscale, BAPQa = Broad Autism Phenotype Questionnaire, Aloof Personality, BAPQpl = Broad Autism Phenotype Questionnaire, Pragmatic Language, BAPQr = Broad Autism Phenotype Questionnaire, Rigidity.

*= *p* < .05, **= *p* < .01, ***= *p* < .001.

In light of the correlation between the value attributed to the approach scenario and the quality of instrumental learning (as indicated by α and w parameters), we wanted to explore whether this could be related to differences observed in response repetition after approach/avoidance feedback between the groups with and without an explicit avoidance strategy. Past research indicates that win-stay responses—i.e., repeating a response after a rewarded choice—are a proxy of an individual’s sensitivity to positive feedback, whereas lose-switch responses—i.e., not repeating a response after a punished choice—attests to an individual’s sensitivity to negative feedback^52–55^. Therefore, we expected to find a correlation between the tendency to repeat the response after approach/avoidance feedback and the learning rate, as well as the w parameter. Since we had a direct measure of the extent to which subjects valued approach and avoidance scenarios, we also correlated the tendency to repeat with the subjective evaluation of those scenarios. Concerning approach feedback, the tendency to repeat a response was indeed negatively correlated with α (ρ = − 0.24, *p* < 0.001) and positively correlated with w (ρ = 0.46, *p* < 0.001) and, importantly, with subjective evaluations (ρ = 0.36, *p* < 0.001). Conversely, concerning avoidance feedback, the tendency to repeat a response was only positively correlated with α (ρ = 0.36, *p* < 0.001) but neither correlated with w (ρ = − 0.02, *p* = 0.75) nor with subjective evaluations (ρ = 0.00, *p* = 0.95).

## Discussion

The current study set out to explore the possibility that the aversive value of threatening (angry) facial displays that one encounters in everyday social contexts can drive uninstructed instrumental CS-avoidance learning. The main result of two online studies on independent large cohorts of participants is that, in the absence of any explicit instruction concerning the presence of threatening expressions, or concerning the necessity to avoid them, at the group level participants spontaneously learned to avoid—i.e., choose the action that maximizes their distance from—individuals displaying anger. Participants subjectively evaluated avoidance feedback as positive and approach feedback as negative. For most participants (approximately two-thirds), learning was not accompanied by the development of an explicit threat-avoidance strategy and was mostly driven by trials in which avoidance was successful. The remaining participants developed an explicit avoidance strategy that led to better learning. In this group, learning was influenced by feedback from both successful and unsuccessful avoidance trials and was associated with more negative evaluations of the social threat scenarios overall. Results from RL models suggest that participants integrated counterfactual information (outcome from the unchosen option) during instrumental CS-avoidance learning. This demonstrates an understanding of the (simple) model of the environment, which was more pronounced in the group that developed an explicit avoidance strategy, even if still present in the group without an explicit strategy.

Research on human defensive behaviors in response to socio-emotional displays mostly focused on species-specific reactions to threat, e.g., freezing^34^, or on stimulus-driven avoidance tendencies^35–37^. If stimulus-driven reactions prevail in the case of highly imminent threat^56^, everyday social behaviors, including defensive behaviors, are more often executed under threat which is less imminent, but see e.g.,^57^, where instrumental mechanisms might predominate. Furthermore, recent research in humans^58,59^ and other animals^60,61^ converge in assigning a more important role to instrumental defensive responses, either under low^62^ or relatively imminent threat^2,10,63^. Consistently with the abovementioned literature, we have previously shown that human approach/avoidance decisions between multiple targets for action in social contexts were influenced by the presence of threat-related facial displays. We suggested that such an influence was mediated by the fact that threat displays increased the value of the action leading to avoidance^32^. We revealed that avoidance decisions did indeed result from rapid and implicit value-based competition between action alternatives, a mechanism sourced in the ventromedial prefrontal and the orbital cortices^31^, for a conceptual replication see^64^, and modulated by changes in participants’ positive affective state^30^.

The abovementioned results strongly support the involvement of instrumental processes in the emergence of avoidance decisions to socio-emotional cues. Nonetheless, the parallel involvement of stimulus-response processes in avoidance decisions cannot be ruled out, since threat-displays were, in every instance, being presented on the screen at the time as subjects made their choice. In contrast, in the present reinforcement learning task, we prevented any involvement of a stimulus-response effect by only displaying neutral individuals at the time of the choice, thereby isolating the instrumental component of avoidance. Past research has already evidenced that emotional facial displays can act as social reinforcers. For instance, it has been demonstrated that individuals can learn to choose actions that lead to the appearance of pleasant rather than unpleasant facial displays^65,66^, or to the disappearance of angry, as opposed to neutral, facial displays^45^. Here we went one step further, by establishing that individuals can learn instrumentally to choose actions that maximize the spatial distance from a threatening individual, while keeping the presence of an angry individual on scene constant across trials. Most importantly, we demonstrated that such learning instantiates spontaneously in a volatile environment, in the absence of any instruction regarding threat avoidance or the structure of the environment, lending greater credence to the possibility that instrumental avoidance learning plays a crucial role in real-life social interactions.

We observed inter-individual differences in instrumental CS-avoidance learning. The subjective value that subjects attributed to action outcomes (approach/avoidance) seemed crucial in accounting for such individual differences. On one hand, in exploratory RL models, not presented in the main text of this work, subjective values assigned in the subjective evaluation task to each possible outcome (approach/avoidance) were entered in the RL models as reward values, fitting quite well participants’ learning (see Supplementary Material, Fig. S4). On the other hand, the more subjects negatively evaluated the approach, but not the avoidance scenarios, the greater their instrumental learning, as indicated by correlations with both α (learning rate) and w (weighting between Q and H values) parameters of the counterfactual model. These results are consistent with the idea that the subjective motivational value associated with the action outcome is a crucial determinant of instrumental avoidance^6^. In line with this, a recent study has shown that the more a stimulus predicting a CS + acquired negative valence throughout the task, the stronger the CS-avoidance^67^. We also observed that response repetition after avoidance feedback (often labelled win-stay) and switch after approach feedback (lose-shift) correlated with both the α and w parameters, confirming that win-stay and lose-switch are proxies of individual sensitivity to positive and negative feedback, respectively^52–55^. Interestingly, the correlation between the subjective value for the approach scenarios and the instrumental learning parameters was subtended by the fact that the more subjects negatively evaluated approach scenarios, the more they tended to switch responses after approach feedback (lose-shift). This nicely parallels animal findings, where the more rats negatively valued punishment, the better their instrumental avoidance learning^47^. Overall, these results indicate that individual differences in avoidance learning might be sourced to individual sensitivity to the negative approach scenarios, which specifically affects the capacity to learn from threat approach feedback.

Heightened negative subjective value might also relate to the development of an explicit avoidance strategy. That minority of participants who reported having used an explicit avoidance strategy throughout the task did have a more negative subjective evaluation of the proposed scenarios and showed better instrumental learning overall. However, most participants did not develop an explicit avoidance strategy, despite showing clear behavioral signs of instrumental avoidance learning on average. In agreement with correlational results, we observed different sensitivity to positive (avoidance) and negative (approach) outcomes between these groups, as revealed by the proportion of response repetition. While both groups displayed higher than chance response repetition after successful avoidance trials (win-stay), only participants who developed an explicit avoidance strategy reduced their proportion of repetition after approach feedback (lose-switch). This confirms that better learners have a heightened capacity to take into account negative feedback to guide their decisions. This also opens the possibility that the group without an explicit avoidance strategy was driven mainly by an appetitive learning mechanism, given that they more often repeated the previous response after avoidance feedbacks, while being at chance level after approach feedbacks. Appetitive motivation facilitates behavior directed toward desirable outcomes, while aversive motivation pushes away from undesirable ones^68^. Both negative reinforcement deriving from escaping threat and positive reinforcement from reaching safety are known to contribute to avoidance learning, but how to precisely disentangle these aversive and appetitive components is still a matter of controversy^1^. In our task, sitting close to the individual that remained neutral might have been made attractive, due to the fact that the other individual turned angry, thus positively reinforcing behavior.

Despite the concordance of both behavioral and subjective data in suggesting that the appetitive component is the main source of learning in the group without an explicit strategy, stronger evidence would be needed to reach a firm conclusion on the nature of the motivational drives (appetitive/aversive) for learning in the two groups. On the one hand, it is questionable that one action option could have been interpreted as absolutely “safe” or “dangerous”, as throughout the task, similarly to two-way shuttling box tasks e.g.,^12,69^, both responses are associated to negative/positive consequences, thanks to probability reversals. On the other hand, differently from two-way shuttling box tasks, within each block there is indeed the possibility for the subject to learn which is the “safe” and the “dangerous” option, opening for the contribution of both appetitive and aversive motivation to learning, which might shift as a function of the development of an explicit avoidance strategy. Still, at least another mechanism could explain learning. The response that leads to threat approach may be perceived as being “punished” by the sudden appearance of the angry individual, thus reducing its frequency at the advantage of the complementary response e.g.,^70^. Thus, we cannot completely exclude that neither positive nor negative reinforcement learning are at place, and that instead a different instrumental mechanism, i.e., social punishment, is involved. One way in which future studies could shed some light on these different possible motivational influences on learning in the present task, would be to measure facial electromyographic activity from the zygomatic and the corrugator muscles, to have a trial-by-trial measure of the perceived valence during feedback phase^71^. This might provide a somehow more direct measure of feedback valuation, to complement post-hoc subjective ratings.

RL model comparisons shed light on another important source of individual difference in learning; the model incorporating a counterfactual learning module best accounted for CS-avoidance behaviors in all subjects, but more clearly for the group who developed an explicit avoidance strategy. Counterfactual learning, which refers to the ability to learn both from the outcomes of the chosen option as well as from forgone outcomes^39^, has proven to be of importance in avoidance learning e.g.,^40^, as well as in value contextualization, enabling symmetrical reward and punishment learning^72^. Counterfactual learning has been compared to model-based (as opposed to model-free) learning, due to the fact that the subject monitors all different possible action possibilities and updates option values according to their predicted outcomes^73^. Interestingly, explicit task structural knowledge has been shown to increase participants use of model-based, rather than model-free, reinforcement learning^74^. In the present task, subjects with an explicit strategy did efficiently integrate counterfactual information, thus behaving more similarly to what would be expected from an instructed group. We can speculate that, as the group with an explicit strategy rated more negatively all social threat scenarios, the overall choice context may have been evaluated as more negative for this group as compared to the other group of subjects, triggering value contextualization and symmetrical learning from both approach and avoidance feedback. It has to be noted that, compared to the simple model, the counterfactual model was slightly better at explaining the data of the group without an explicit strategy as well (small effect size), indicating that, at least for some participants, a representation of the model of the environment was present, even in absence of an explicit goal to avoid or of a reportable representation of the probabilistic structure of the task.

The current study is not without its limitations. First, most participants (n = 145, 119 without explicit strategy, 26 with explicit strategy; see Figs. 2, 3) had a non-significant binomial test against chance level on the mean proportion of hits. This suggested that a large proportion of individuals did not exhibit a clear behavioral sign of learning on average throughout the task, which might somehow limit generalizability of the conclusions concerning spontaneous avoidance learning in social contexts. However, as showed in supplementary analyses (Fig. S5 and Table S6), this group of seemingly non-learners took in to account the previous feedback to adjust behavior. Accordingly, they repeated the response given at the previous trial significantly more often after avoidance than approach feedbacks on average. Such feedback-driven response repetition proves that they were sensitive to the most recent action outcome, as the rest of the group. This of course does not eliminate the fact that their average performance remained at chance level, replicating in a social context the well-known strong inter-individual variability in instrumental avoidance learning. The sources of individual differences in learning also relate to the second limitation of the study, which is the absence of strong correlations between questionnaires of affective/personality measures and behavior or model parameters—there was indeed a small correlation between the Behavioral Inhibition System and the w parameter (see^75^ for similar findings). This constrains, for the moment, the generalizability of our conclusions to pathological social avoidance, such as that seen in anxiety disorders or autism. A possible reason for this result might reside in the nature of our sample, which was selected from the general population, without targeting any specific clinical group, where variability in anxiety or autism scores are likely to be reduced. Another possible explanation resides in the very nature of the task, which consists of what is called “low-cost” avoidance, in that it only requires a button press, without, for example, either a physical effort, or a cost in terms of reward loss. While “low-cost” avoidance tasks have proven effective for studying CS-avoidance in humans e.g.,^76^, their capacity to account for pathological avoidance has been criticized^5,6,77^, due to the fact that they do not take into account that pathological avoidance is often accompanied by an omission of reward (e.g., not attending a concert in order to avoid being surrounded by people), which renders it costly. We hope to have provided some insight on some of the sources of inter-individual variability and encourage future research to explore it further. Moreover, testing the present task on clinical groups to evaluate whether the inclusion of action costs is needed to tap into pathological mechanisms of active CS-avoidance.

## Conclusion

The present experiment has demonstrated that the negative motivational value associated with anger expressions suffices to drive the emergence of spontaneous instrumental defensive actions, aimed at CS-avoidance. In most subjects, instrumental active avoidance was independent from any form of explicit understanding of the action-outcome contingencies, suggesting that spontaneous instrumental defensive strategies might play a role in our ability to navigate our ever changing and complex social environment. In line with Amodio^78^, the classical dual-process view in social cognition, which advocates that non-declarative (implicit) behaviors consist solely of automatic stimulus-stimulus or stimulus-response associations, and that instrumental processes only intervene to exert cognitive control, is probably too simplistic. Implicit behavioral tendencies, biases, and attitudes toward others are more likely the product of a multitude of learning processes, including instrumental learning, which guarantee flexibility and adaptation to volatile environments^78^. Our results further support that individual differences in active instrumental avoidance in social settings are strongly dependent on the subjective value attributed to threatening expressions, and in particular, to how we feel when we find ourselves in proximity of threatening individuals. The construction throughout life, for instance via direct experience or observation, of our unique representation of what others’ emotional expressions mean to us likely plays a role in shaping what we expect from those others, and ultimately drives the way we spontaneously navigate our social worlds.

## Methods

### Participants

278 participants participated in the experiment. 64 participants were not included in the final sample after the application of the following exclusion criteria: (1) not having completed the task (n = 3); (2) having reported problems with the task display on screen, in particular concerning a time delay between the display of the two halves of the main task scene (n = 43) or the fact that the green tick was not properly displayed on the chosen chair (n = 10) (see task section), or both problems together (n = 3); (3) declaring having been diagnosed with a neurological or psychiatric disease (n = 1); (4) having responded to less than 90% of trials in the allotted time (n = 1); (5) having anticipated more than 15% of the total of valid responses (response anticipation was defined as a response time inferior to 100 ms) (n = 3). The final sample included 214 participants (140 declared females, declared age: m = 34.9, sd = 13.2, min/max = 18/75, declared handedness: 191 right, 21 left, 2 ambidextrous) who had normal or corrected-to-normal vision and no history of neurological or psychiatric disorders. The experimental protocol was approved by the Comité d’Evaluation Ethique de Institut National de la Santé et de la Recherche Médicale (INSERM - IRB00003888 - N° 120-689bis) and was carried out in accordance with the Declaration of Helsinki. Participants provided informed consent and received a compensation for their participation to the study. The mean payment was £11.13 per hour (average experiment duration was 37.2 min).

### General procedure

Participants were recruited online via the Prolific platform (www.prolific.ac). The experiment was coded in JavaScript, using the library jsPsych 6.1.0^79^ and was hosted on Pavlovia’s servers (www.pavlovia.org). After providing their consent, participants filled in a short survey collecting their demographic information (gender, age, handedness and level of education) as well as the short version of the State Trait Anxiety questionnaire (STAI-S)^80^. Following this initial phase, participants performed the reinforcement learning task (from now on “main task”), which lasted approximately 15 min, and was preceded by task instructions, and by a 1 min training session. At the end of the main task, participants answered several task-related questions (see Supplementary Material). The main task was followed by the subjective evaluation task, which began with task instructions and a 1 min training session. Participants were then debriefed regarding the post-test difficulty, the realistic aspect of the scene and the quality of the display. Finally, they filled in the trait scale of the short version of the State Trait Anxiety questionnaire (STAI-T)^80^, the Broad Autism Phenotype Questionnaire (BAP-Q)^81^ and the Behavioral Activation/Inhibition Scales (BAS/BIS)^82^.

### Reinforcement learning task

The main task (Fig. 1) was adapted from previous studies^30–32^.

#### Stimuli

Subjects were presented with a scene representing a waiting room with four chairs, where the two middle chairs were occupied by two individuals and the two outer chairs were empty (See Fig. 1). Each scene was the composite of one template female or male hemi-scene (photograph depicting either one female or one male sitting next to an empty chair) juxtaposed to its mirrored version, on which faces were superimposed. Ten (five males, five females) fixed pairs of identities (RadBound Faces Database^83^) that were matched for gender as well as perceived trustworthiness and threat traits were used^32^. Both neutral and angry expressions were used for the task, with angry expressions corresponding to the strongest level of intensity used in previous studies from our lab^31,32^. The fixed pairs of identities as well as the identities’ position in the scene were counterbalanced and each specific scene was presented 15 times in a random order (with the constraint that one stimulus could not be presented more than twice in a raw). This resulted in 300 trials (2 sexes × 5 pairs × 2 positions × 15 repetitions).

#### Experimental procedure

Participants were instructed that the experiment consisted of a series of decisions, and that they had to indicate where they would prefer to sit in the waiting room, while maintaining fixation on the fixation cross throughout the trial. Participants were invited to make spontaneous free choices and were informed that there were no wrong choices. Each trial started with a grey screen, displayed for 500 ms, then a fixation cross appeared for a time varying between 500 and 700 ms. Fixation’s position was in the middle of the two (to be presented) individuals’ faces, at the level of the eyes. After the fixation, the scene appeared and remained on the screen until a valid response (consisting in a left or right button press within the given time) was registered, or until a maximum time of 1500 ms, in the case of no response. If no response was registered, a message saying ‘TOO SLOW’ was displayed. To respond, participants used their keyboard, pressing “S” for sitting on the left chair or “L” for the right chair. After participant response, a visual mask consisting of a random permutation of the pixels of the previously presented scene was displayed for 200 ms, and feedback was given immediately after (500 ms). This feedback represented the same scene, with a green tick symbol superimposed on the chosen chair, indicating the participant’s position in the scene. Importantly, either the individual close to or far from the chosen chair changed expression from neutral to angry. Therefore, the feedback for the participant consisted in finding themselves seated far from (avoidance) or close to (approach) the angry individual. At each trial, one response (e.g., sit on the right chair) was associated with an 80% probability of avoidance in the feedback phase and to a 20% probability of approach. The other response (e.g., sitting on the left chair) was associated with the complementary probability (20% avoidance, 80% approach). Importantly, action-outcome probabilities were reversed on average every 25 trials, to ensure continuous goal-directed learning and to discourage habit formation. Accordingly, the 300 trials were divided into 12 blocks of different trial length, presented in a randomised order for each participant, sampling from the same uniform distribution (three blocks of 20 trials, three blocks of 24 trials, three blocks of 26 trials and three blocks of 30 trials). It should be noted that all action-outcome contingencies were hidden to participants, who were never given the impression they were to learn anything and to whom neither emotions, nor approach/avoidance were ever mentioned. Pauses to allow participants to rest were introduced at the beginning of the 5th and 9th blocks. A training session was performed before the main experiment to familiarize participants with the task. The training consisted of 2 separated sequences of 10 trials, using 4 pairs of individuals (2 pairs of females), which were different from those used in the main experiment. During feedback, both individuals remained neutral. After the first 10 trials, participants were informed of their percentage of valid responses and were asked to do their best to maximize it if their score was below 80% accuracy.

### Subjective evaluation task

#### Stimuli

In the reinforcement learning task, at each trial, participants received feedback contingent upon their chosen action, consisting of either an avoidance scenario (i.e., green tick indicating their position in the scene far from the individual displaying anger) or an approach scenario (i.e., green tick close to the individual displaying anger). In the subjective evaluation task, we aimed at obtaining an explicit subjective evaluation of all possible feedback scenarios. We therefore presented stimuli consisting of the same waiting room scenes, with all the possible pairs of individuals, each displaying either a neutral or angry expression, with the green tick indicating participant's position either far from (avoidance scenario) or close to (approach scenario) the angry individual. This resulted in 40 trials (10 pairs × 2 anger position × 2 participant's position). While in the reinforcement learning task the respective position (i.e., left vs. right) of each actor in the scene was also randomized, here, as we were not interested in obtaining different subjective judgements dependent upon actor position, we randomly assigned actors' positions.

#### Experimental procedure

Each trial started with a grey screen displayed for 500 ms, then a fixation cross was superimposed on the upper center of the grey screen for a time varying between 800 and 1200 ms. Once the fixation cross disappeared, the scene was presented for 1000 ms, followed by a screen with a visual analog scale. Contrary to the main task, the fixation cross was not maintained during the presentation of the scene, in order to allow for free visual exploration. Participants were subsequently requested to indicate the degree to which they would have liked to find themselves in the situation depicted in the scene on a visual analog scale ranging from ‘NOT AT ALL’ to ‘EXTREMELY’. To respond, participants had to click on their cursor and move it along the scale before validating their response using the “continue” button that appeared after they had moved the cursor. By clicking on the “continue” button, the participant ended the trial. A quick training session was performed before the post-test to familiarize participants with the task. The training session consisted of 4 trials, using 2 pairs of individuals (1 pair of females), which were different from those used in the main experiment. The green tick was superimposed twice on the right chair and twice on the left chair. The orders of stimuli presentation and tick side were randomized.

### Statistical analyses

Concerning the reinforcement learning task, the main dependent variable was the probability of “hits”, namely the proportion of trials in which participants choose to sit on the chair which maximized the probability of avoidance in the feedback phase over the total number of trials. The probability of response repetition, namely the proportion of trials in which participants repeated the same response given in the previous trial (trial n − 1), was also analyzed to further characterize learning.

Concerning the subjective evaluation task, the main dependent variable was the subjective evaluation of each scenario as indicated by participants’ response on a scale coded on a continuum between 0 and 100, corresponding respectively to ‘NOT AT ALL’ and to ‘EXTREMELY’.

#### General mixed models (GLMs)

All GLMs were run on R 4.0.4 (2021–02-15)^84^, using RStudio 1.3.1093^85^ using the “glmer” function from the lme4 packag ^86^. We also used other R packages for handling data^87–89,89,90^, for generating plots^91–93^ and tables^94,95^.

For all mixed models presented below, we took subjects as a random variable and kept a maximal random effect structure^96^, by including all by-subject random intercepts and random slopes (where present) in the model. Where present, main effects and interactions including between-subjects variables where only included as fixed effects^96^.

#### Reinforcement learning task: hits

To evaluate whether participants displayed any evidence of learning over the course of the entire task, we compared the probability of hits to a chance level of 0.5, by conducting a mixed logistic regression, with the probability of hits as a dependent variable and the intercept as a predictor measuring difference from chance^48^.

To test learning in time within each block after a reversal of the action-outcome probability, we conducted a mixed logistic regression, with the probability of hits as a dependent variable and the hyperbole of the trial number as a within-subject independent variable (hyperbolic function: ${\text{y}} = 1{ } - { }1/\left( {1 + {\text{x}}} \right)$). This analysis was restricted to the first 20 trials after each reversal since block lengths differed. Trial numbers were re-coded as ranging from 0 to 19, to have a meaningful intercept estimate (corresponding to the moment of probability reversal) and facilitate model fitting.

#### Reinforcement learning task: repetition

To investigate whether participants learned differently after approach or avoidance feedback, for each trial after the first trial of the experiment, we conducted a mixed logistic regression, with the probability of repetition as a dependent variable and the feedback obtained at trial n − 1 (0 = approach, 1 = avoidance) as a within-subject independent variable.

We also wanted to account for possible differences in the subjective evaluation of feedback scenes. As the subjective evaluation task provided us with an estimate of the subjective value that each subject attributed to each possible feedback seen in the main task, we paired real feedbacks obtained at each trial of the reinforcement learning task with the corresponding subjective value estimate given in the subjective evaluation task. Of note, we acknowledge that these subjective value estimates could not take into account possible variation of subjective value throughout the main task, as they were collected in a separate task, as explained in the methods. As of the second trial, we conducted a mixed logistic regression, with the probability of repetition as a dependent variable and the estimate of the subjective evaluation of the feedback obtained at trial n − 1 as a within-subject independent variable. Subjective ratings were re-coded as ranging from 0 to 1, by dividing them by 100, to facilitate model fit.

#### Subjective evaluation task

We conducted a mixed linear regression, with the subjective evaluation (0–100) as a dependent variable and the scenario (0 = approach, 1 = avoidance) as a within-subject independent variable.

#### Explicit versus non-explicit avoidance strategy

Finally, to account for the presence of an explicit avoidance strategy, all previous models were also run with the addition of the main effect of strategy (0 = non-explicit, 1 = explicit) as a between-subject variable as well as of the interaction of strategy with the other variables.

#### Reinforcement learning (RL) models

To further characterize learning, we ran variations of simple reinforcement learning models, which try to estimate each action option’s expected reward (Q), in each choice context, in order to choose the action that maximizes Q^49,50^.

In our task the context (or state) is always the same, thus the value for the chosen option (c) at trial t is updated with the following delta rule

1 $${\text{Q}}_{{{\text{t}} + 1}} \left( {\text{c}} \right) = {\text{Q}}_{{\text{t}}} \left( {\text{c}} \right) + \upalpha \updelta _{{{\text{c}},{\text{t}}}}$$

where α is the learning rate and δ_c_ is the prediction error term for the chosen option, calculated as

2 $$\updelta _{{\text{c,t}}} = {\text{R}}_{{\text{c,t}}} - {\text{Q}}_{{\text{t}}} \left( {\text{c}} \right)$$

where R_c_ is the obtained reward. Reward coding was 1 for avoidance feedback, and 0 for approach feedback.

Due to the peculiarity of this task, where the action choice which maximizes reward (i.e., threat avoidance) remained the same over the course of an entire block before probability reversal, we chose to slightly complexify the base RL model, to account for the tendency to repeat actions previously performed (i.e., perseveration). To do so we adapted a model proposed by Miller and colleagues^51^ and added, for each action option, a habit value H updated at each trial as follows

3 $$\left\{ {\begin{array}{*{20}c} {{\text{H}}_{{{\text{t}} + 1}} \left( {\text{c}} \right) = {\text{H}}_{{\text{t}}} \left( {\text{c}} \right) + {\upalpha }_{{{\text{hab}}}} (1 - {\text{H}}_{{\text{t}}} \left( {\text{c}} \right))} \\ {{\text{H}}_{{{\text{t}} + 1}} \left( {\text{u}} \right) = {\text{H}}_{{\text{t}}} \left( {\text{u}} \right) + {\upalpha }_{{{\text{hab}}}} (0 - {\text{H}}_{{\text{t}}} \left( {\text{u}} \right))} \\ \end{array} } \right.{ }$$

We acknowledge that, in our task, a true habit would probably not have the time to develop over the course of a block, therefore the α_hab_ parameter can be considered as a measure of response perseverance, i.e., the simple tendency to repeat previously performed responses.

Q value and H value were then merged in a global action value (D) through the following formula

4 $${\text{D}}_{{\text{t}}} \left( {{\text{c}},{\text{u}}} \right) = {\text{wH}}_{{\text{t}}} { }\left( {{\text{c}},{\text{ u}}} \right) + \left( {1 - {\text{w}}} \right){\text{ Q}}_{{\text{t}}} \left( {{\text{c}},{\text{ u}}} \right)$$

where w is a weighting parameter which tends to 1 when the D value is predominantly determined by response perseverance and tends to 0 when it is driven by goal-directed processes. In the model, Q and D values were initialized at 0.5 and H value at 0.

Subject choice behavior, i.e., the probability to choose one action a over the other action b, was modeled via a softmax function

5 $${\text{P}}_{{\text{t}}} \left( {\text{a}} \right) = \frac{1}{{1 + {\text{e}}^{{\left( {{\text{D}}_{{\text{t}}} \left( {\text{b}} \right) - {\text{D}}_{{\text{t}}} \left( {\text{a}} \right)} \right)*\upbeta }} }}$$

with β being the inverse temperature parameter. β values tending to infinite make actions that differ in value to be selected with greater difference in probability. β values tending to zero render the action selection more and more equiprobable.

We were interested in knowing whether the learning was counterfactual, i.e., whether or not subjects updated both the value of the chosen and the unchosen options at each trial. We therefore compared a simple model, which did not update the value of the unchosen option after each choice

6 $${\text{Q}}_{{{\text{t}} + 1}} \left( {\text{u}} \right) = {\text{Q}}_{{\text{t}}} \left( {\text{u}} \right)$$

to counterfactual models, which we did in the following way

7 $${\text{Q}}_{{{\text{t}} + 1}} \left( {\text{u}} \right) = {\text{Q}}_{{\text{t}}} \left( {\text{u}} \right) + \upalpha \updelta _{{\text{u,t}}}$$

where ${\updelta }_{{{\text{u}},{\text{t}}}}$ is based on ${\text{R}}_{{{\text{u}},{\text{t}}}} = { }1 - {\text{ R}}_{{{\text{c}},{\text{t}}}}$.

#### Model fitting, comparison, and simulation

Parameter optimization was conducted by minimizing the negative log-likelihood using Matlab’s fmincon function^97^, initialized at starting points of 1 for the inverse temperature and 0.5 for the other parameters. To constrain parameter fitting toward plausible values, the following prior distribution were used to weight the negative log-likelihood during model fitting: (1) a gamma distribution with a shape parameter *k* = 1.2 and a scale parameter θ = 5, for the inverse temperature parameter and (2) a beta distribution with shape parameters α = 1.1 and β = 1.1 for all the other parameters. Using the optimal individual parameters, model estimates of action choice were generated, trial-by-trial, 100 times and averaged.

To check that models fit the data better than chance, we ran a Friedman test^98^ on model log-likelihood to compare a random model, giving random choices (50% probability of choosing right or left), to the simple and the counterfactual models. We used Wilcoxon paired tests to further compare models and determine whether the simple or the counterfactual model best fit the data. We also tested the ability of each model to reproduce the real data, by running Spearman correlations between the mean proportion of hits in real and simulated data. Finally, for the winning model, we ran unpaired Wilcoxon tests to compare parameters between subjects with and without an explicit avoidance strategy.

#### Correlations between model parameters and subjective measures

Finally, we computed Spearman correlation between subject’s best fitting parameters for the winning model and questionnaire measures, to test whether instrumental avoidance learning was associated with affective or personality features. We also correlated model parameters with the mean subjective value attributed by each subject to approach and avoidance scenarios in the subjective evaluation task.

## Data and code availability

All data and code are available at the https://osf.io/7usfe/ online repository. For any further information, please contact Rocco Mennella.

## Supplementary Information

Supplementary Information.
