# Supplementary material for: Spontaneous instrumental avoidance learning in social contexts
Source: Sci Rep. 2022 Oct 20;12:17528. doi: 10.1038/s41598-022-22334-6 (PMC9585085; doi:10.1038/s41598-022-22334-6)
Supplement: Supplementary file 1 — Supplementary Information. [file 41598_2022_22334_MOESM1_ESM.pdf]

Spontaneous instrumental avoidance learning in social contexts

Rocco Mennella, Sophie Bavard, Inès Mentec, Julie Grèzes

Supplementary Methods

Pilot Study: methods

Participants

62 French volunteers participated in the pilot study. 6 participants were not included in the final sample after the application of the same exclusion criteria as in the main task: 1) not having completed the task (n = 3); 2) having reported problems with the task display on screen, in particular reporting a time delay between the display of the two halves of the main task scene (n = 5); 3) having responded to less than 90% of trials in the given time (n= 1). The final sample included 56 participants (35 declared females, declared age: m = 28.6, sd = 10.7, min/max = 18/61, declared handedness: 46 right, 10 left) who had normal or corrected-to-normal vision and no history of neurological or psychiatric disorders. The experimental protocol was approved by the Comité d'Evaluation Ethique de Institut National de la Santé et de la Recherche Médicale (INSERM - IRB00003888 - N° 120-689bis) and was carried out in accordance with the Declaration of Helsinki. Participants provided informed consent and did not receive a compensation for their participation to the study.

General procedure

Same as the main experiment except for the following: 1) the whole procedure was in French, the sample being French speaking; 2) the subjective evaluation task was not included, so the subjective evaluation of each possible approach/avoidance scenario is not available for the pilot study; 3) the questions for the debriefing were arranged slightly differently from the main experiment and did not include the questions 10 and 14 (see Subjects' debriefing: list of questions). Also, the level of education was not demanded, and the option “ambidextrous” for the question about handedness was not provided.; 4) participants completed a short version of the BAPQ instead of the full version [1].

Reinforcement learning task and experimental procedure

Same as the main study.

Statistical analyses

We run the same mixed logistic models (including the effect of strategy) as in the main experiment. In the absence of the subjective evaluation task, contrary to the main task, we could neither run the model on the subjective evaluation data, nor the one on the probability of response repetition predicted by feedback's subjective value. Results can be found in Table S3 and data visualization in Figure S2.

Subjects' debriefing: list of questions

After reinforcement learning task

1. Did you find the task difficult? (0: not at all, 10: very difficult)
2. Did you manage to fix your attention on the cross throughout the task? (0: not at all; 10: absolutely)
3. Did you notice anything on the faces of the individuals of the scenes? If so, describe what you saw in few words. (If you didn't notice anything, write 'nothing')
4. Did this influence your choices? If so, how? (If not, write 'no')
5. Did you notice any emotion on the faces of the individuals? If so, describe what you saw in few words.

After subjective evaluation task

6. Did you find the task difficult? (0: not at all, 10: very difficult)
7. To what extent did the situation seem real to you? (0: not at all; 10: absolutely)
8. How would you rate the display's quality during both experiments? (0: mediocre; 10: excellent)
9. Did you notice any display offset between the two halves of the image for some trials (considering both experiments)?
10. Did the green tick appear on one of the seats (considering both experiments)?
11. How many hours do you sleep on average each night?
12. How many hours did you sleep last night?
13. Did you consume any alcohol or drugs yesterday? If so, what and how much?
14. Have you ever been diagnosed with a neurological or psychiatric disease?
15. We are facing a global health crisis. Do you think this affected the way you performed the task? If yes, describe in few words how.

Supplementary Figures

Figure S 1. Results from the subjective evaluation task. Left: results for the whole sample. Right: results split by strategy with the group without an explicit avoidance strategy in blue and the one with an explicit strategy in violet. Bars' height and black points represent the mean. Error-bars represent confidence intervals 95% for the normal distribution. Shaded points represent single subjects' means.

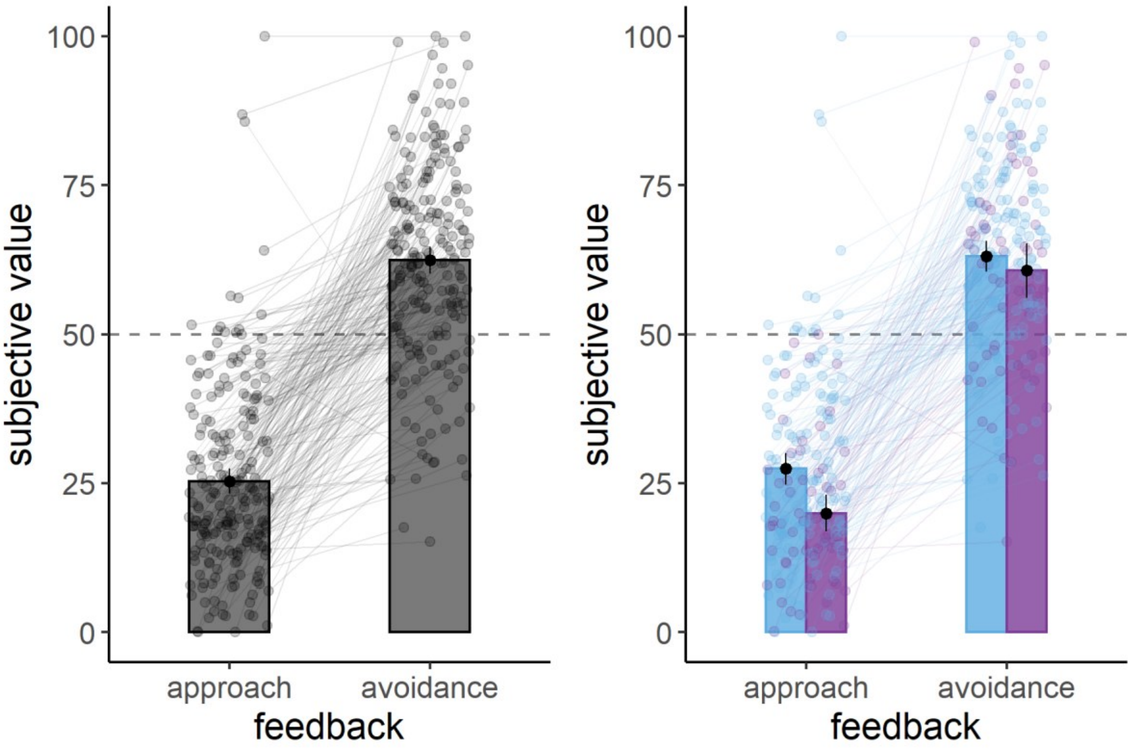

Figure S 2. Summary of behavioral results for the pilot study. Left: mean proportion of hits throughout the task for the group without an explicit avoidance strategy in blue and the group with an explicit strategy in violet (the same color code applies for the remaining sections of the figure). Red points represent the mean and error-bars represent the confidence intervals at 95% for the normal distribution. Shaded points represent single subjects' means and grey's tone reflects whether, within each subject, the binomial test against chance (0.5) is significant (dark grey) or not (light grey). Right top: mean proportion of hits across the first 20 trials over blocks of stable action-outcome contingency (trial 1 = reversal trial). Points represent means within trial and error-bars represent confidence intervals at 95% for the normal distribution. The fitted curves represent the best fit (and 95% confidence interval) for the same hyperbolic function used in the mixed linear models (see Methods). Right bottom: mean proportion of action repetition following either an approach or an avoidance feedback. Black points represent means and error-bars represent confidence intervals 95% for the normal distribution. Shaded points represent single subjects' means.

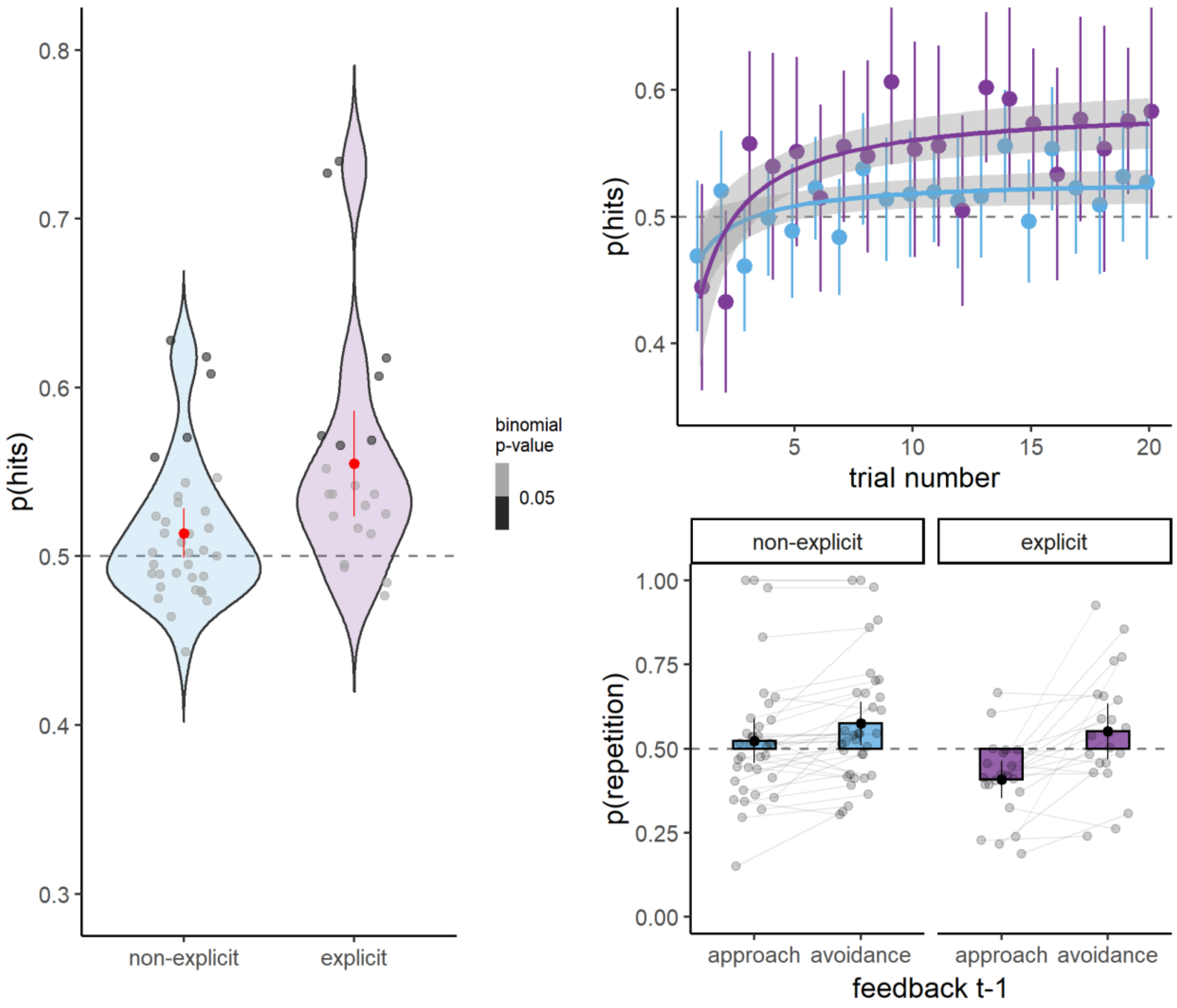

Figure S 3. Summary of how the subjects experienced the reinforcement learning task. The figure represents the frequency distribution for subjects' responses to questions 8 (left top), 7 (left bottom), 2 (right top) and 1 (right bottom) from the final debriefing.

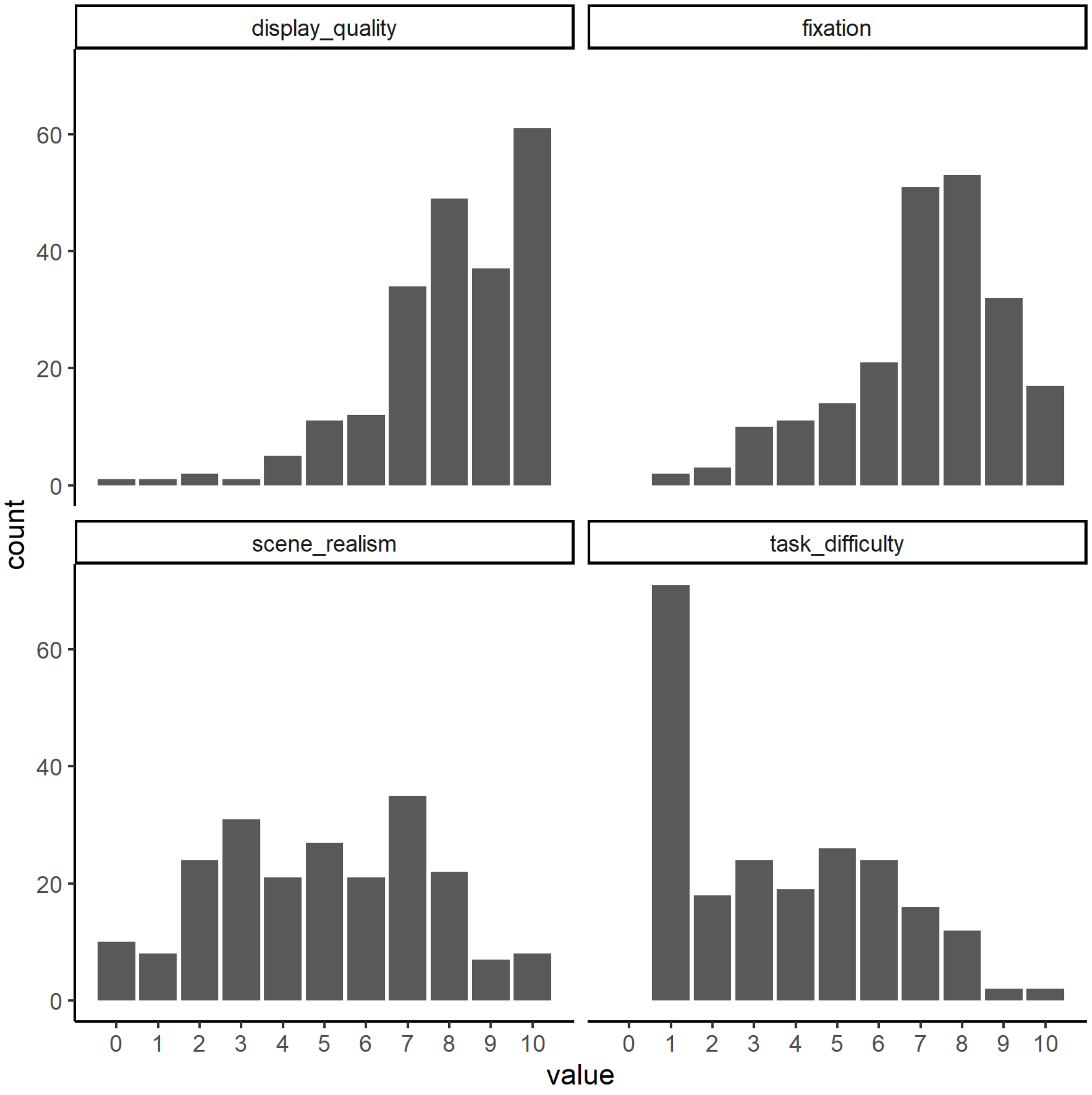

Figure S 4. Results of RL models in which we used as reward value (R) the subjective value estimate provided by the subjects in the subjective evaluation task. As for the GLM (model 4, see Methods), at each trial we entered in the RL model the subjective value obtained in the subjective evaluation task which corresponded to the real feedback obtained at each trial of the reinforcement learning task. Values were scaled from 0 to 1. All information provided in the legend for Fig. 3 in the paper applies to Fig. S4. The figure highlights the differences between the group without an explicit avoidance strategy (light blue) and the group with an explicit strategy (violet). Prediction from the simple and the counterfactual reinforcement learning models are in blue and turquoise, respectively (solid lines for the group without an explicit strategy and dotted lines for the one with explicit strategy). Blue and turquoise points represent means of simulations, and the fitted curve in the left top graph represents the best fit (and 95% confidence interval) for the same hyperbolic function used in the mixed linear models, fitted on simulated data. Right bottom, correlation between real and simulated mean hit proportions for the simple and the simulated models, as a function of the presence (violet) or the absence (light blue) of an explicit avoidance strategy.

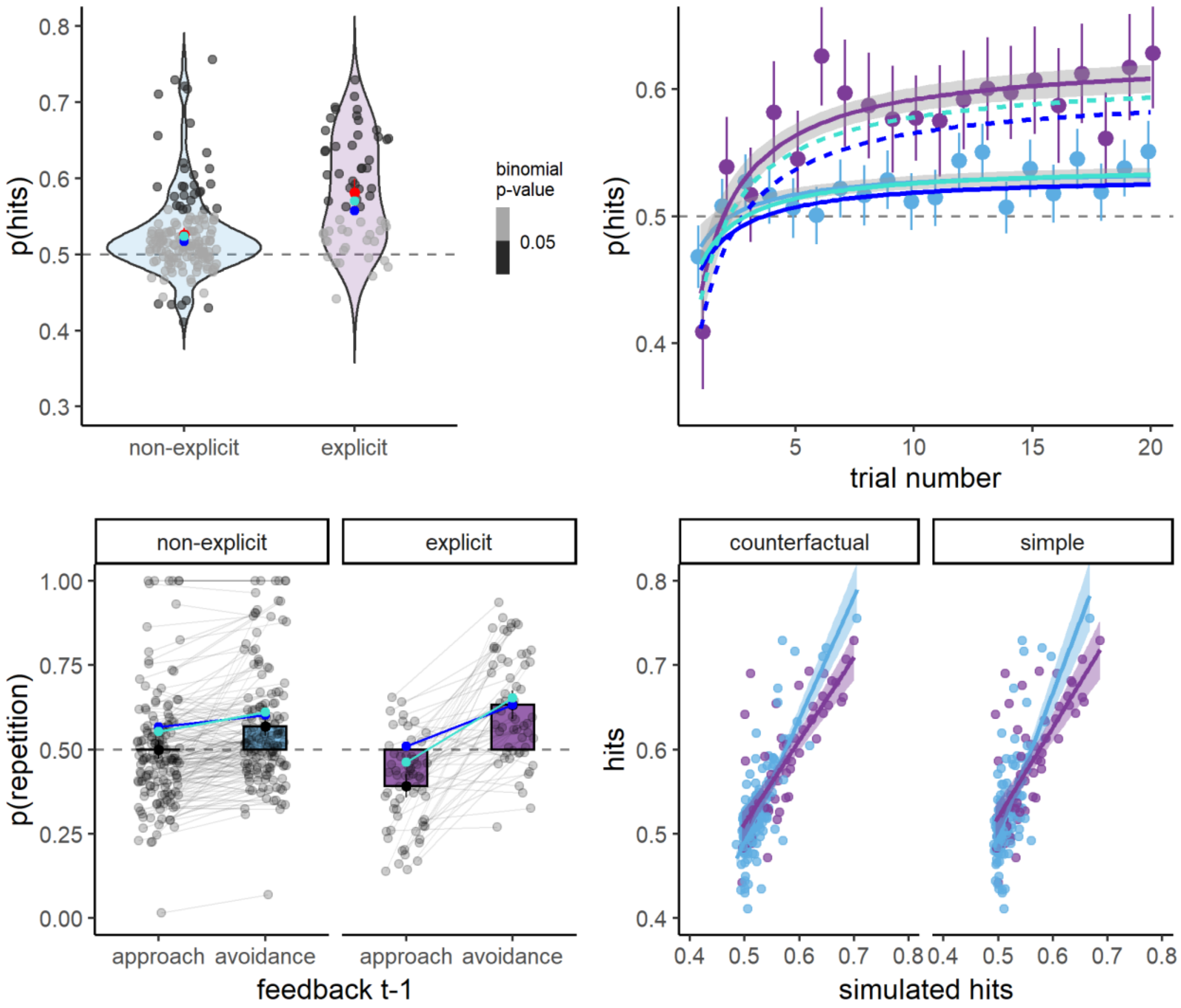

Figure S 5 GLM results in seemingly non-learners in the mean hit proportion. Most subjects (n = 145, 119 without explicit strategy, 26 with explicit strategy) had a non-significant binomial test against chance level on the mean proportion of hits throughout the task (Figure 2, top right). To investigate whether signs of learning emerge also in this sub-sample of seemingly non-learners, we plotted the proportion of repetition for the main study, as a function of the objective feedback at t – 1 (top) or of the subjective value attributed to this feedback (bottom). See Table S6 for statistical details.

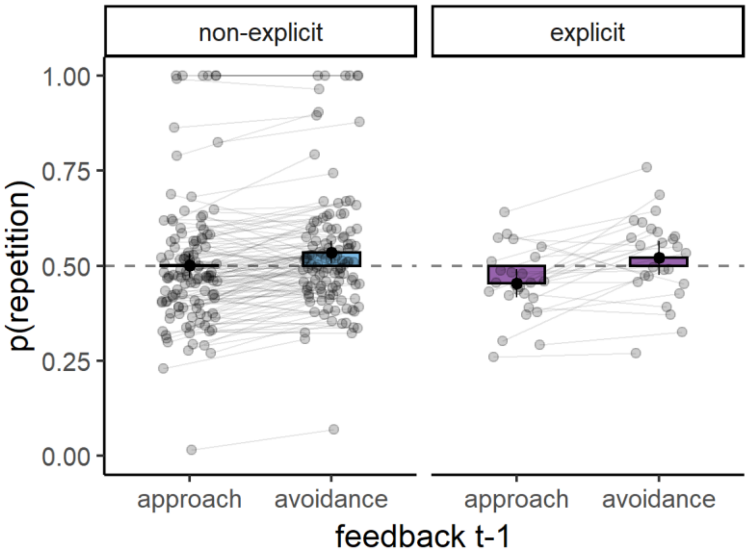

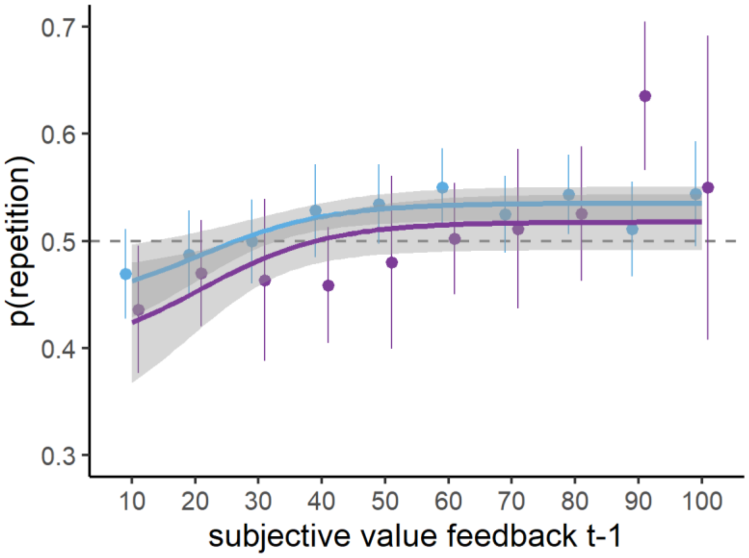

Supplementary Tables

Table S1: Mixed logistic models results for the subjective evaluation task

	**model 1 : subjective value**			**model 2 : subjective value**		
Predictors (across models)	Estimates	CI	p	Estimates	CI	p
Intercept	25.34	23.24 – 27.44	**<0.001**	27.43	25.01 – 29.85	**<0.001**
scenario (avoidance)	37.05	33.97 – 40.13	**<0.001**	35.63	32.00 – 39.25	**<0.001**
strategy (explicit)				-7.45	-12.02 – -2.88	**0.001**
scenario (avoidance) * strategy (explicit)				5.07	-1.77 – 11.91	0.146
**Random Effects**						
σ^2	259.47			259.47		
τ_{00}	232.10 subject			221.94 subject		
τ_{11}	502.90 subject.scenario (avoidance)			500.17 subject.scenario (avoidance)		
ρ_{01}	-0.69 subject			-0.68 subject		
ICC	0.49			0.49		
N	214 subject			214 subject		
Observations	8560			8560		
Marginal R^2 / Conditional R^2	0.403 / 0.695			0.409 / 0.696		

Table S2: Subjects' debriefing, full report

subject	strategy	something on faces	influence on choice
1	non-explicit	Frowns	No
2	non-explicit	Some looked angry	sometimes
3	explicit	Anger / annoyance	Yes if they looked angry after I sat next to them, I would sit on the opposite side the next time
4	non-explicit	Nothing	No
7	non-explicit	Some were frowning looking annoyed	Not really
9	explicit	At first all the faces were neutral but after selecting a seat one of the faces would turn to a scowl.	I felt less comfortable re-selecting the same seat on my next choice after I chose one that caused the person next to the free seat to scowl.
10	non-explicit	Angry looks	NO
12	non-explicit	I noticed in my peripheral vision that they had appeared to be angry after the decision. Before the decision I think they had neutral expression	No
14	non-explicit	Scowling in some	Not consciously
15	non-explicit	Frowning	No
17	non-explicit	Angry faces	No
18	non-explicit	Nothing	NO
19	explicit	Anger	Yes I tried to remember the grumpy people & not sit by them as much as possible
20	non-explicit	I think they were initially passive then after I made my choice became negative	No
21	non-explicit	They went from neutral to frowning	Tried to find a pattern but couldn't
22	explicit	Blank face before then one of them would look unhappy / frown after seat selection was made	Yes - I would choose the other seat next time!

23	explicit	Anger / frustration	I chose to sit next to the person who looked less angry
24	non-explicit	Angry	Sometimes
26	explicit	Yes either a blank expression one of anger one of disgust	Yes I tried to sit next to the person who didn't get upset with me sitting next to them. I probably shouldn't have been looking.
29	non-explicit	Some were annoyed when you sat beside them	Yes as after a while you were more able to tell if they would be annoyed from memory
30	non-explicit	Blank then after the decision they changed	No
33	explicit	Annoyance and anger	Yes it made me steer away from some of them
35	non-explicit	Blank faces which turned into anger/frustration	No
36	non-explicit	Angry	Yes
37	explicit	Some seemed almost disgusted or confused	Sometimes if I noticed it would make me move to the other side the next time rather than recognising the person's face and avoiding them
38	explicit	Anger	Yes I didn't want to sit with them
39	non-explicit	Some looked a bit frowny - I'd have sat next to them just to be contrary :)	I wanted them to but there wasn't enough time for that to happen and I wasn't sure if it was right to do that so no it didn't really influence my choices.
40	explicit	I noticed disconfort or anger.	I started to remember who changed expression and avoided to sit near those individuals.
41	explicit	Yes they changes expressions	Yes if the expression was angry I did select the other option in the next round
43	non-explicit	Some looked angry and the faces changed just after I made the selection	slightly but I didn't have a chance to look as I was supposed to keep fixed on the x
44	non-explicit	Changes went from neutral to angry	No
48	non-explicit	They looked bored	No
49	explicit	Yes - emotions of resting to emotions of disappointment/disapproval	Yes - when I did catch sight of an individuals expression I choose the most amiable one.
50	explicit	Sometimes I noticed annoyance before but definitely noticed annoyance after making my decisions.	Yes I tried to avoid sitting next to people who would scowl.

51	explicit	Annoyance / frustration	Occasionally if I saw people were annoyed for example I may choose to sit on the other side
52	non-explicit	Annoyed or angry	I don't think so!
53	non-explicit	Disgruntled a bit angry or shocked. The rest of the faces were neutral.	I don't think it conciously influenced my choices because there was so little time to think just click.
54	non-explicit	they would change to become angry sometimes	No
55	non-explicit	Anger/annoyed	No
56	non-explicit	Yes after a selection you could see one person's face expression change	No
57	non-explicit	Angry	No
61	non-explicit	Normally the individuals had pretty blank expressions on their faces but I noticed that sometimes the expressions changed to something like anger or irritation.	At the start I think may have made it harder to focus on the cross but then due to the speed in which choices had to be made and how random the changes were I don't think it consciously influenced my choices.
62	non-explicit	Sad/angry face on one person after choosing a seat.	No.
63	explicit	Some of the emotions I thought I saw included anger confusion and possibly sadness	Yes prefer not to sit next to people who looked angry
64	non-explicit	Sometimes the faces got angry	Yes
66	explicit	Yes some people looked angry or upset	I might be less likely to choose the people who frowned but couldnt always see in the short time and with staying focused on the cross
67	non-explicit	Sadness or anger	No
68	explicit	Anger disgust	Yes tended to sit on the other side after seeing a rude face
69	non-explicit	Angry expression	No
70	non-explicit	Cross/grumpy on some	No
72	non-explicit	After I made my choice one of the individuals would have an angry face	No
73	explicit	Yes some looked angry.	yes. I usually choose not to sit near them.

74	explicit	Emotions changed to more negative/angry after decision	Tried not to but think it made me avoid that side
75	explicit	Anger? annoyance? disgust?	Yes if I selected the left side and the person changed expression then I would select the right side for my next decision
76	explicit	Neutral (both) and then annoyed (one) after selection	Yes I tried to sit on the opposite side of the person I thought might get angry looking
78	non-explicit	Anger / disgust / frustration	No
79	non-explicit	Angry or stern	Yes
80	non-explicit	Smiles or fowns	Dont think so
81	explicit	Anger	Yes I tried to choose not to be with the angry person
82	non-explicit	They looked angry	No
83	non-explicit	The expressions went from neutral to angry in some cases	No
85	explicit	Some faces were passive others were angry or alarmed.	I think that the facial expressions on the previous choice influenced me to sit in the other seat instead.
86	non-explicit	Blank looking	No
87	non-explicit	Were angry when clicked on.	No
88	explicit	After selection one face would appear disgruntled/angry	On occasion I couldn't help but determine that one individual would look more welcominga nd so would choose to sit beside them. If it got this wrong however I did feel a little uncomfortable with my selection
89	non-explicit	Some looked more welcoming; some appeared as sombre.	Initially this may have affected a subsequent choice but that tendency to be influenced later diminished.
92	non-explicit	Yes I noticed anger in their faces	Yes but I cannot explain why
94	explicit	Nothing	Yes chose other person if noticed in time
95	non-explicit	Some looked angry sometimes	No
100	non-explicit	After my selection some looked angry	No

102	explicit	Boredom	Yes I'd like to sit next to a relaxed cheerful person
103	non-explicit	Anger/Disgust I know it so well	I tried to select them, and pick them out.
104	non-explicit	They got angry/upset.they frowned.	I dont know may be subconsciously yes. sometimes I liked the persons face even if they got angry before.
105	non-explicit	Nothing	No
106	non-explicit	Yes some of them did not like it that I wanted to sit next to them	No
107	non-explicit	Nothing	I don't know. yes?
108	explicit	Different expressions neutral or frowning	Yes as once I began to notice I would rather sit next to the individual with a neutral expression as they seemed more approachable
110	explicit	Angry frustrated sad	If they were neutral I stuck to that side if angry I swapped sides
111	non-explicit	I would call it a grimaces howing dislike and distaste	I don't think it did as it was impossible to keep track of who was grimacing at me (which was randomised beforehand anyway)
112	explicit	Frowning	Yes it made me not want to sit by them if they frowned
114	non-explicit	Angry annoyed	Sometimes made me change who I chose
115	non-explicit	Anger	No
116	non-explicit	They got angry when I chose to sit next to them or someone else.	Yes
117	non-explicit	Yes frowning anger nervousness	Yes
119	non-explicit	Nothing	No
120	explicit	Some individuals had expressed negative emotions after the choice	Yes I tried to avoid those whose facial expressions have previously changed
121	non-explicit	No emotion at first then after clicking one of them would change to a frown.	Unsure if it did or not.
122	explicit	Yes - some were happy and others were solemn. This changed when the choice had been made.	Yes as I would have felt more confident sitting by them.

123	non-explicit	Varied - some no expression some angry some with screwed up/contorted faces	No
124	explicit	Anger disgust.	I was trying to figure out a pattern so as not to sit next to an angry face but I could not work it out.
125	non-explicit	As previous answerone of the faces changed to an angry expression	No
126	non-explicit	Yes it changed after choosing	No
127	non-explicit	Angry or annoyed	No
128	non-explicit	The faces were neutral before selecting a seat and one of them became upset or angry afterwards.	No
129	non-explicit	Faces changed when chosen/not chosen	No
131	non-explicit	Some of them had slightly warmer expressions but the experiment was too fast to dwell on it and mostly only noticeable after the choice was made	No
132	non-explicit	Anger	No
133	non-explicit	Grumpy	No
134	explicit	Yes I saw neutral faces but also angry (frowning) and some silly faces	Sometimes it would make you pick a different side if your eyes drifted from the cross and you noticed this
135	explicit	Anger and irritation	I felt if I was making quicker choice immediately following - it felt like I was more likely to go the opposite way
136	non-explicit	Yes - some were angry	No
137	non-explicit	Annoyed after choosing one	No
138	non-explicit	Yes annoyance	I don't think so
139	non-explicit	Some of them seemed angry sometimes	I think some of them looked a bit angrier
140	non-explicit	I could notice a disappointed look	No
143	non-explicit	Some had a slight smile some looked very serious	It didnt at first then I found it did then I made a conscious decision that it wouldnt

145	non-explicit	Yes some grimaced/frowned	No there was no time to process/remember the expression
146	explicit	Disgust	Yes I did not want to sit next to them later
147	non-explicit	Anger annoyance	Maybe? I tried to be impartial but felt drawn to certain faces
148	non-explicit	After I chose one become serious/miserable	No
149	non-explicit	anger or annoyance	No
150	non-explicit	Yes some individuals were serene and others were upset.	No
151	non-explicit	Some appeared sad or angry	No
152	explicit	I saw one of the pair look grumpy / angry after I had made my selection	Yes it made me think I had to try and choose the non-angry person
153	non-explicit	I think some of the images changed expression after I had selected an image	Yes
155	explicit	Anger displeasure	Yes influenced me to press the other seat position on the next turn.
157	non-explicit	Some faces weren't as friendly had moody faces	No
158	non-explicit	Only noticed it after my choice on the reset screen - where sometimes they looked crossed.	No
159	non-explicit	Annoyed cross disappointed	No
161	non-explicit	Sadness	no
162	non-explicit	Just angry	Norecuasse they changed after selection
163	non-explicit	Disgust or anger could not tell which exactly it was.	I don't think I made a conscious decision to choose differently because of the faces but it certainly will have had some sort of subconscious influence in future decisions.
164	non-explicit	Grumpy or irritated	No
165	explicit	Yes. Angry fed up & fear	Yes. I did not want to sit next to an angry person
167	non-explicit	Sometimes they frowned	No

168	non-explicit	When scene is first shown both have neutral expressions. The person you chose to sit next to looked annoyed in scene with tick	No
169	explicit	Stern grumpy	Yes I would pick the person who looked more friendly
170	non-explicit	One frowned or grimaced after each selection	No
173	non-explicit	Yes - negative emotions. Disgust/anger.	Probably a bit.. even though I had to respond quickly it probably influenced me if the person had previously glared at me for choosing them or maybe even not choosing them.
174	non-explicit	They got angry	No
175	non-explicit	When I was selecting the seat the faces didn't show any emotions however after I select the seat one of the faces in each pair changed - they had a grimace or a frown.	No
176	non-explicit	Nothing	A little maybe
177	explicit	Anger and annoyance	Yes I went for the ones that looked happier.
178	explicit	Some of them seemed cross that I sat next to them	If I remembered they frowned before I didn't sit by them
179	explicit	Some looked annoyed some frowning some more friendly than others.	Yes tried to avoid faces that changed.
180	non-explicit	Astonishment anger disappoimtment	No
181	non-explicit	Anger/disapproval and neutral/resigned	No not consciously
182	non-explicit	Yes , some faces changed emotion after selection of seat	No
184	non-explicit	Anger and annoyance after I chose	Yes I was trying to figure out who wanted me to sit next to them based on that but it seemed random
185	non-explicit	Yes some had nothing some looked disgusted	No
186	non-explicit	Some people looked unwelcoming a little bit ticked off	Not really I tried to follow your instructions to look at the cross.
187	non-explicit	Angry faces sometimes after I pushed the button.	No I don't think so.

188	non-explicit	After choosing a side some of their facial expressions changed- creased eyebrows	No
189	non-explicit	Frowns	No
190	non-explicit	Often irritated if I chose to sit next to them	Not consciously
191	non-explicit	Some seemed annoyed and some had a menacing expression.	No
192	explicit	Only that after my selection one of them would appear to become unhappy.	In the final block I avoided faces I thought were changing but which ones did didn't seem consistent so by the very end this factor influenced me a bit less.
193	non-explicit	Not before choosing only after	No
197	non-explicit	Many had a cross or worried/nervous expression	No
198	explicit	Before the choices the individuals looked calm and after that some of then looked angry	Yes i was trying to sit next to the person that wouldn't turn angry
199	non-explicit	Mostly no emotion sometimes I noticed they were angry	It did not influence my choices but it did distract me and at times I questioned if it was the same looking people who's faces changed
200	explicit	Some people looked angry and others confused.	I believe it did. Most people wouldn't want to sit next to someone who is angry or not a nice person.
202	non-explicit	Angry and sad	No
203	non-explicit	The expressions seemed to show sadness but I didn't focus on them enough to reaaly be sure.	It did influence me a few times at the beginning but then I became more focused on the cross and didn't notice the faces.
204	non-explicit	Happy, serious, indifferent	No
205	non-explicit	Only after the decision cant tell their emptions while focusing on the cross	Don't know... it must have influenced my decision on a subconscious level but I didnt use that feedback consciously
206	non-explicit	Sometimes people looked cross that I had either chosen the seat next to them or the other seat!	No I wasn't aware that it did.
207	non-explicit	Hurt	No
208	non-explicit	Some looked more serious others looked relaxed	No

209	non-explicit	Some frowned after you picked the seats next to themsome frowned if you picked the other seat	No
210	non-explicit	Nothing	No
212	non-explicit	Some were angry	No
213	non-explicit	Like unhappiness or disapproval a sense of screwing up of facial features or pulling displeased faces.	I felt like it should or at times thay I should try to avoid them doing this but I couldn't affect any change and continued to use instinct despite the thoughts of the above aspect crossing my mind.
214	explicit	Anger	Sometimes it would put me off sitting there
215	explicit	Grumpy angry unhappy	I think I tried to avoid the faces that looked less happy
216	explicit	Yes they looked mad or angry or upset	Yes I tried to select the seat where their expression wouldn't change
217	non-explicit	Annoyed irritated smiling	No
218	non-explicit	I saw that sometimes the individuals had a neutral expression at first and then this changed to an angry grimacing expression.	No
220	non-explicit	I think a negative emotion on some of the men	No i don't think so
221	non-explicit	Especially after when it clicked up with the tick I saw grimaces etc and sometimes I felt there was almost contempt in the face	Yes that influenced my choice when I noticed but being so quick and concentrating on the cross sometimes I just noticed the hair
223	explicit	Smile, anger, irritation	Sometimes I chose friendlier face, sometimes it was to late
224	explicit	Blank and then angry	I tried memorising which faces got angry more and tried choosing ones that won't
225	non-explicit	Not before making my choice	Tried to remember the ones that did not grimace
226	non-explicit	Worried engry, cross, calm	No
227	non-explicit	Nothing	Yes it made me feel wary of what their face might look like if I wasn't looking at the cross
228	non-explicit	Yes angry or annoyed perhaps	No
229	non-explicit	Yes they looked angry	Yes

230	non-explicit	Neutral emotion before selection this would change to a negative emotion (annoyed angry confused) after I selected	No. I am not making a judgement on their personality. If it's a 50:50 choice between two seats there is no difference to me so how they react does not affect me and is irrelevant to me
232	non-explicit	Some were angry or annoyed	Not really because I couldnt see their expressions while I was looking at the X
233	explicit	Some were angry some were neutral	I think in some cases it did as I didn't choose to sit next to the angry person
234	non-explicit	Yes their emotions are change after I made my decisions	No
235	non-explicit	Angry and misunderstanding	In the beginning yes but then I realize even I chose them or the other person they sometimes would put a weird face and sometimes not so I just stopped paying attention to them
236	explicit	Anger	I didnt want to sit near them
237	explicit	Happiness in some of them some almost looked fed up	Yes I was drawn more towards people who were smiling slightly or looked friendly.
238	non-explicit	Mostly concern and unhappiness	No
239	non-explicit	Annoyance anger	I'm not sure as I was trying to focus on the x rather than trying to avoid those faces.
240	non-explicit	They changed to grumpy faces	No
241	non-explicit	Anger	Hair colour
242	non-explicit	Nothing	No
243	non-explicit	They repeatedly changes emotions to bitterness	Sometimes yes because faces blended over time
244	non-explicit	Anger or irritation appeared on one face after the selection	No
245	non-explicit	They kept changing throughout each trial	No
246	explicit	I noticed negative emotions anger suspicion.	I was trying to remember which person was upset the previous time and where I clicked so that I could click differently but it was hard to do.
250	non-explicit	They seemed to me as they were bored	No
251	explicit	Some were angry when I chose to sit near them	Yes I didn't want to sit near the angry one

252	explicit	Yes frowning mostly	Occasionally I would avoid choosing to sit next to the ones who frowned the most.
253	non-explicit	Ye sthe individuals became angry after I chose my seat sometimes	No
254	non-explicit	Yes sometimes the emotion changed to an angry or annoyed look	No
255	non-explicit	Scowling	No
256	non-explicit	Yes some people looked at ease others looked uncomfortable	No
257	non-explicit	Annoyance?	Yes tried to remember which ones frowned more
260	explicit	Anger or annoyed	I think so I wouldn't want to sit with someone who looked angry
261	non-explicit	I noticed smiles grimaces frowns	No
263	non-explicit	Their expressions sometimes showed anger	No
264	explicit	Yes some faces were angry/annoyed/unfriendly	Yes if I noticed that when I sat next to a person their face was negative I tended to steer clear if possible
265	non-explicit	Yes they smiled	I'm not sure, it was such a fast experiment that it may have subconsciously influenced my choice but I didn't have time to think enough to allow it it.
267	explicit	Sometimes one of the people would show an angry or frustrated expression after I had made my choice	Possibly a little - I would always go for my first instinct but the expression did stick in my mind and sometimes I noticed myself avoiding that side.
268	non-explicit	Their expressions changed	No
269	non-explicit	Expressions sometimes changed after making the choice where to sit	No
270	explicit	I noticed some of them where angry and some looked worried or scared.	I think it did as sometimes the face would stay with me and I wouldn't choose them next time. but I did choose fast and I could of forgot the face so I'm not sure
272	non-explicit	Some individuals where happy and some where angry	No
273	non-explicit	I think I registered some scowls some neutral looks and maybe smiles...	Because of the time limit I can't say I consiously made a decision but maybe subconsciously...

274	non-explicit	Some went grumpy	No
275	non-explicit	They were responding with various forms of negative emotions often outright contempt and or disgust.	It did at times. Especially if I got someone who reacted particularly negatively. This tended to be more with the women. One might as well chance ones arm on the other side! The blokes I tended to just shrug off. On other occasions I just ignored it and sat where I wanted. I am exhausted atm so this was for most of the survey.

Table S3: Mixed logistic models on pilot data

	**model 1 : hits**			**model 2 : hits**			**model 3 :repetition**		
Predictors (across models)	Odds Ratios	CI	p	Odds Ratios	CI	p	Odds Ratios	CI	p
Intercept	1.06	0.98 – 1.14	0.126	0.86	0.70 – 1.05	0.145	1.30	0.91 – 1.84	0.151
strategy (explicit)	1.18	1.05 – 1.33	**0.005**	0.84	0.60 – 1.18	0.314	0.52	0.29 – 0.93	**0.027**
hyp(trial)				1.29	0.98 – 1.68	0.067			
hyp(trial)*strategy				1.46	0.94 – 2.27	0.090			
feedback t-1 (avoidance)							1.34	1.08 – 1.67	**0.008**
feedback t-1*strategy							1.42	1.01 – 2.00	**0.042**
**Random Effects**									
σ^2	3.29			3.29			3.29		
τ_{00}	0.03 subject			0.11 subject			1.08 subject		
τ_{11}				0.29 subject.hyperbole(trial_num)			0.31 subject.rew_prec_factavoidance		
ρ_{01}				-0.95 subject			0.08 subject		
ICC	0.01			0.01			0.28		
N	56 subject			56 subject			56 subject		
Observations	16472			13172			16278		
Marginal R^2 / Conditional R^2	0.002 / 0.012			0.004 / 0.015			0.022 / 0.297		

Table S4: Group without an explicit strategy. Spearman's correlations between counterfactual model parameters and subjective measures

	SVapp	SVav	STAIt	STAIs	BIS	BASd	BASr	BASf	BAPQa	BAPQpl	BAPQr
alpha	-0.17*	0.05	-0.02	0.05	0.01	0	0.03	0	-0.11	-0.13	-0.02
alpha_hab	0.11	-0.01	-0.01	0	-0.07	-0.03	0.05	0	0.02	0.12	0.08
beta	0.08	-0.21*	-0.04	0.01	0.01	0.04	0	0.02	0.1	0.05	0.01
w	**0.3*****	-0.01	-0.1	-0.04	-0.13	0.17*	0.05	0.03	0.03	-0.04	-0.07

Notes: * = p < .05, ** = p < .01, *** = p < .001. Correlations in bold survive FDR correction for multiple comparisons. SVapp = subjective value for approach scenarios, SVav = subjective value for avoidance scenarios, BIS = Behavioral Inhibition System, BASd = Behavioral Activation System, Drive subscale, BASr = Behavioral Activation System, Sensitivity to Reward subscale, BASd = Behavioral Activation System, Funseeking subscale, BAPQa = Broad Autism Phenotype Questionnaire, Aloof Personality, BAPQpl = Broad Autism Phenotype Questionnaire, Pragmatic Language, BAPQr = Broad Autism Phenotype Questionnaire, Rigidity

Table S5: Group with an explicit strategy. Spearman's correlations between counterfactual model parameters and subjective measures

	SVapp	SVav	STAIt	STAIs	BIS	BASd	BASr	BASf	BAPQa	BAPQpl	BAPQr
alpha	-0.14	-0.01	0.19	0.06	0.17	-0.11	-0.15	0.04	0.04	-0.05	-0.05
alpha_hab	0.24	0.13	0.04	-0.11	0.02	-0.07	0	0.09	0.02	0.03	0.01
beta	-0.16	-0.1	0.15	0.2	0.11	0.02	-0.05	0.08	-0.01	0.05	-0.12
w	0.25	-0.14	-0.06	0.03	-0.25	0.12	0.02	0.13	-0.31*	-0.01	-0.4**

Notes: * = p < .05, ** = p < .01, *** = p < .001. Correlations in bold survive FDR correction for multiple comparisons. SVapp = subjective value for approach scenarios, SVav = subjective value for avoidance scenarios, BIS = Behavioral Inhibition System, BASd = Behavioral Activation System, Drive subscale, BASr = Behavioral Activation System, Sensitivity to Reward subscale, BASd = Behavioral Activation System, Funseeking subscale, BAPQa = Broad Autism Phenotype Questionnaire, Aloof Personality, BAPQpl = Broad Autism Phenotype Questionnaire, Pragmatic Language, BAPQr = Broad Autism Phenotype Questionnaire, Rigidity

Table S6: Mixed logistic models. Extra GLM analyses on seemingly non-learners

	model 1 : repetition			model 2 : repetition		
Predictors (across models)	Odds Ratios	CI	p	Odds Ratios	CI	p
Intercept	1.14	0.94 – 1.39	0.190	1.06	0.87 – 1.30	0.544
feedback t-1 (avoidance)	1.17	1.10 – 1.24	**<0.001**			
strategy (explicit)	0.72	0.45 – 1.16	0.175	0.69	0.43 – 1.11	0.126
feedback t-1 (avoidance)*strategy (explicit)	1.13	0.99 – 1.29	0.067			
subjective value feedback t-1				1.38	1.22 – 1.55	**<0.001**
subjective value feedback t-1*strategy (explicit)				1.31	1.01 – 1.70	**0.043**
**Random Effects**						
σ^2	3.29			3.29		
τ_{00}	1.17 $_{subject}$			1.16 $_{subject}$		
τ_{11}	0.03 $_{subject.rew\_prec\_factavoidance}$			0.10 $_{subject.subjValue\_prec}$		
ρ_{01}	-0.15 $_{subject}$			-0.18 $_{subject}$		
ICC	0.26			0.26		
N	145 $_{subject}$			145 $_{subject}$		
Observations	42047			42047		
Marginal R^2 / Conditional R^2	0.004 / 0.264			0.005 / 0.260		

Supplementary References

1. Stojkovic, I., Ducic, B., Kaljaca, S. & Djordjevic, M. Construction and psychometric evaluation of a short form of the Broad Autism Phenotype Questionnaire. Psihologija **51**, 243–258 (2018).
